# Supplementary material for: Temporal Dynamics of Gene Expression During Metamorphosis in Two Distant Drosophila Species
Source: Genome Biol Evol. 2025 May 27;17(6):evaf100. doi: 10.1093/gbe/evaf100 (PMC12202745; doi:10.1093/gbe/evaf100)
Supplement: evaf100_Supplementary_Data [file evaf100_supplementary_data.zip › Supplementary tables.pdf]

GO	name	GO type	p-value FDR *D. melanogaster*	p-value FDR *D. virilis*
GO:0003677	DNA binding	molecular function	3.09E-101	1.22E-56
GO:0003676	nucleic acid binding	molecular function	3.75E-81	7.39E-69
GO:0051252	regulation of RNA metabolic process	biological process	6.14E-90	6.56E-37
GO:0019219	regulation of nucleobase-containing compound metabolic process	biological process	1.23E-89	6.56E-37
GO:2000112	regulation of cellular macromolecule biosynthetic process	biological process	1.00E-87	6.56E-37
GO:0010556	regulation of macromolecule biosynthetic process	biological process	2.00E-87	6.56E-37
GO:0006355	regulation of transcription, DNA-templated	biological process	2.47E-86	6.56E-37
GO:1903506	regulation of nucleic acid-templated transcription	biological process	2.47E-86	6.56E-37
GO:2001141	regulation of RNA biosynthetic process	biological process	2.47E-86	6.56E-37
GO:0031326	regulation of cellular biosynthetic process	biological process	3.98E-84	6.56E-37
GO:0009889	regulation of biosynthetic process	biological process	1.59E-83	6.56E-37
GO:0010468	regulation of gene expression	biological process	8.05E-85	2.36E-34
GO:0060255	regulation of macromolecule metabolic process	biological process	1.26E-80	1.10E-34
GO:0051171	regulation of nitrogen compound metabolic process	biological process	4.66E-79	2.76E-35
GO:0080090	regulation of primary metabolic process	biological process	1.20E-76	2.76E-35
GO:0031323	regulation of cellular metabolic process	biological process	4.05E-76	1.18E-35
GO:0043565	sequence-specific DNA binding	molecular function	6.71E-81	1.77E-28
GO:1901363	heterocyclic compound binding	molecular function	5.25E-55	3.66E-53
GO:0019222	regulation of metabolic process	biological process	2.03E-74	1.06E-33
GO:0097159	organic cyclic compound binding	molecular function	3.04E-54	3.66E-53
GO:0050794	regulation of cellular process	biological process	5.79E-71	9.07E-30
GO:0140110	transcription regulator activity	molecular function	1.17E-81	1.92E-16
GO:0050789	regulation of biological process	biological process	2.58E-69	1.54E-28
GO:0005488	binding	molecular function	1.19E-45	6.04E-51
GO:0003700	DNA-binding transcription factor activity	molecular function	6.13E-78	2.13E-17
GO:0065007	biological regulation	biological process	1.19E-61	2.02E-27
GO:0005634	nucleus	cellular component	3.71E-62	9.95E-27
GO:0032502	developmental process	biological process	1.03E-81	0.01191084257
GO:0048856	anatomical structure development	biological process	1.53E-72	0.009876830148
GO:0098772	molecular function regulator	molecular function	3.69E-59	1.15E-09
GO:0005515	protein binding	molecular function	7.40E-36	5.23E-17
GO:0046983	protein dimerization activity	molecular function	6.90E-32	9.78E-15

GO:0043231	intracellular membrane-bounded organelle	cellular component	3.39E-26	2.96E-20
GO:0043227	membrane-bounded organelle	cellular component	8.82E-25	3.43E-20
GO:0043229	intracellular organelle	cellular component	2.58E-29	1.86E-15
GO:0043226	organelle	cellular component	3.70E-29	1.86E-15
GO:0046982	protein heterodimerization activity	molecular function	9.60E-23	1.07E-07
GO:0032993	protein-DNA complex	cellular component	2.27E-20	1.54E-09
GO:0044815	DNA packaging complex	cellular component	8.97E-20	3.92E-10
GO:0006325	chromatin organization	biological process	8.24E-24	1.51E-05
GO:0000786	nucleosome	cellular component	4.19E-18	1.40E-09
GO:0016043	cellular component organization	biological process	7.68E-24	4.02E-03
GO:0071840	cellular component organization or biogenesis	biological process	5.92E-24	9.94E-03
GO:0003682	chromatin binding	molecular function	2.65E-22	2.32E-02
GO:0034728	nucleosome organization	biological process	5.12E-17	8.97E-06
GO:0006334	nucleosome assembly	biological process	3.32E-16	8.97E-06
GO:0071824	protein-DNA complex subunit organization	biological process	8.96E-15	8.97E-06
GO:0065004	protein-DNA complex assembly	biological process	3.79E-14	8.97E-06
GO:0003674	molecular_function	molecular function	1.78E-03	4.55E-14
GO:0005694	chromosome	cellular component	4.38E-11	1.49E-03
GO:0022402	cell cycle process	biological process	2.39E-11	0.009955903567
GO:0007166	cell surface receptor signaling pathway	biological process	3.74E-09	7.96E-05
GO:0006270	DNA replication initiation	biological process	1.94E-05	2.28E-08
GO:0051276	chromosome organization	biological process	2.46E-09	3.23E-04
GO:0006260	DNA replication	biological process	2.14E-05	8.16E-08
GO:0042555	MCM complex	cellular component	5.46E-05	3.01E-06
GO:0006259	DNA metabolic process	biological process	9.74E-05	8.97E-06
GO:0022607	cellular component assembly	biological process	2.81E-07	1.42E-02
GO:0007275	multicellular organism development	biological process	2.38E-06	3.00E-02
GO:0007167	enzyme linked receptor protein signaling pathway	biological process	4.75E-06	3.93E-02
GO:0034622	cellular protein-containing complex assembly	biological process	2.10E-03	8.15E-04
GO:0071103	DNA conformation change	biological process	1.66E-03	2.05E-03
GO:0090304	nucleic acid metabolic process	biological process	9.85E-04	6.05E-03
GO:0004713	protein tyrosine kinase activity	molecular function	1.53E-03	5.14E-03
GO:0043933	protein-containing complex subunit organization	biological process	2.62E-03	6.04E-03

GO:0065003	protein-containing complex assembly	biological process	6.10E-03	0.002620579673
GO:0007165	signal transduction	biological process	0.04665196958	0.006072749481
GO:0006357	regulation of transcription by RNA polymerase II	biological process	4.88E-86	
GO:0000981	DNA-binding transcription factor activity, RNA polymerase II-specific	molecular function	9.64E-77	
GO:1990837	sequence-specific double-stranded DNA binding	molecular function	3.48E-76	
GO:0048869	cellular developmental process	biological process	9.76E-76	
GO:0000976	transcription regulatory region sequence-specific DNA binding	molecular function	5.36E-73	
GO:0001067	regulatory region nucleic acid binding	molecular function	6.52E-73	
GO:0003690	double-stranded DNA binding	molecular function	1.55E-72	
GO:0000977	RNA polymerase II transcription regulatory region sequence-specific DNA	molecular function	1.56E-71	
GO:0009653	anatomical structure morphogenesis	biological process	8.20E-69	
GO:0048513	animal organ development	biological process	4.06E-67	
GO:0010558	negative regulation of macromolecule biosynthetic process	biological process	2.73E-61	
GO:2000113	negative regulation of cellular macromolecule biosynthetic process	biological process	2.73E-61	
GO:0031327	negative regulation of cellular biosynthetic process	biological process	7.68E-60	
GO:0009890	negative regulation of biosynthetic process	biological process	4.02E-59	
GO:0048523	negative regulation of cellular process	biological process	1.55E-58	
GO:0048519	negative regulation of biological process	biological process	1.56E-57	
GO:0000978	RNA polymerase II cis-regulatory region sequence-specific DNA binding	molecular function	5.66E-56	
GO:0000987	cis-regulatory region sequence-specific DNA binding	molecular function	6.52E-55	
GO:0010629	negative regulation of gene expression	biological process	7.14E-55	
GO:0051253	negative regulation of RNA metabolic process	biological process	4.10E-54	
GO:0045892	negative regulation of transcription, DNA-templated	biological process	2.75E-53	
GO:1902679	negative regulation of RNA biosynthetic process	biological process	2.75E-53	
GO:1903507	negative regulation of nucleic acid-templated transcription	biological process	2.75E-53	
GO:0045934	negative regulation of nucleobase-containing compound metabolic process	biological process	8.27E-53	
GO:0010605	negative regulation of macromolecule metabolic process	biological process	6.57E-50	
GO:0007389	pattern specification process	biological process	2.82E-47	
GO:0009892	negative regulation of metabolic process	biological process	1.15E-46	
GO:0051172	negative regulation of nitrogen compound metabolic process	biological process	1.43E-46	
GO:0031324	negative regulation of cellular metabolic process	biological process	1.85E-45	
GO:0030154	cell differentiation	biological process	8.80E-45	
GO:0000122	negative regulation of transcription by RNA polymerase II	biological process	8.88E-44	

GO:0051254	positive regulation of RNA metabolic process	biological process	3.96E-38	
GO:0003002	regionalization	biological process	6.77E-37	
GO:0045893	positive regulation of transcription, DNA-templated	biological process	6.77E-37	
GO:1902680	positive regulation of RNA biosynthetic process	biological process	6.77E-37	
GO:1903508	positive regulation of nucleic acid-templated transcription	biological process	6.77E-37	
GO:0050793	regulation of developmental process	biological process	8.18E-37	
GO:0045935	positive regulation of nucleobase-containing compound metabolic process	biological process	1.80E-36	
GO:0010628	positive regulation of gene expression	biological process	2.90E-36	
GO:0048522	positive regulation of cellular process	biological process	5.10E-36	
GO:0048731	system development	biological process	8.94E-35	
GO:0010557	positive regulation of macromolecule biosynthetic process	biological process	3.63E-34	
GO:0048518	positive regulation of biological process	biological process	3.81E-34	
GO:0045595	regulation of cell differentiation	biological process	7.42E-34	
GO:0045944	positive regulation of transcription by RNA polymerase II	biological process	3.88E-33	
GO:0010604	positive regulation of macromolecule metabolic process	biological process	6.22E-32	
GO:0009887	animal organ morphogenesis	biological process	1.11E-31	
GO:2000026	regulation of multicellular organismal development	biological process	1.20E-31	
GO:0051239	regulation of multicellular organismal process	biological process	1.40E-31	
GO:0006928	movement of cell or subcellular component	biological process	1.92E-31	
GO:0009891	positive regulation of biosynthetic process	biological process	2.51E-31	
GO:0031328	positive regulation of cellular biosynthetic process	biological process	2.51E-31	
GO:0097485	neuron projection guidance	biological process	7.22E-30	
GO:0009893	positive regulation of metabolic process	biological process	1.16E-29	
GO:0051173	positive regulation of nitrogen compound metabolic process	biological process	6.67E-29	
GO:0007411	axon guidance	biological process	2.53E-28	
GO:0031325	positive regulation of cellular metabolic process	biological process	3.61E-28	
GO:0050767	regulation of neurogenesis	biological process	7.67E-28	
GO:0060284	regulation of cell development	biological process	4.09E-27	
GO:0001709	cell fate determination	biological process	4.25E-27	
GO:0009888	tissue development	biological process	2.06E-26	
GO:0051960	regulation of nervous system development	biological process	5.76E-26	
GO:0032501	multicellular organismal process	biological process	1.90E-25	
GO:0048468	cell development	biological process	4.29E-25	

GO:0009886	post-embryonic animal morphogenesis	biological process	2.01E-23	
GO:0045165	cell fate commitment	biological process	4.40E-23	
GO:0001708	cell fate specification	biological process	1.70E-21	
GO:0002009	morphogenesis of an epithelium	biological process	4.77E-21	
GO:0030182	neuron differentiation	biological process	4.77E-21	
GO:0022414	reproductive process	biological process	2.19E-20	
GO:0048729	tissue morphogenesis	biological process	3.50E-20	
GO:0035295	tube development	biological process	4.97E-20	
GO:0007423	sensory organ development	biological process	2.05E-19	
GO:0061061	muscle structure development	biological process	4.15E-17	
GO:0035107	appendage morphogenesis	biological process	4.56E-17	
GO:0035239	tube morphogenesis	biological process	4.95E-17	
GO:0007417	central nervous system development	biological process	6.07E-17	
GO:0007419	ventral cord development	biological process	1.71E-16	
GO:0045664	regulation of neuron differentiation	biological process	2.06E-16	
GO:0035114	imaginal disc-derived appendage morphogenesis	biological process	2.15E-16	
GO:0048859	formation of anatomical boundary	biological process	4.22E-16	
GO:0060429	epithelium development	biological process	6.32E-16	
GO:0048812	neuron projection morphogenesis	biological process	1.30E-15	
GO:0048858	cell projection morphogenesis	biological process	1.30E-15	
GO:0120039	plasma membrane bounded cell projection morphogenesis	biological process	1.30E-15	
GO:0032989	cellular component morphogenesis	biological process	1.49E-15	
GO:0032990	cell part morphogenesis	biological process	1.49E-15	
GO:0048867	stem cell fate determination	biological process	1.85E-15	
GO:0051093	negative regulation of developmental process	biological process	2.07E-15	
GO:0048598	embryonic morphogenesis	biological process	2.32E-15	
GO:0022412	cellular process involved in reproduction in multicellular organism	biological process	5.96E-15	
GO:0045596	negative regulation of cell differentiation	biological process	6.07E-15	
GO:0031491	nucleosome binding	molecular function	6.21E-15	
GO:0051128	regulation of cellular component organization	biological process	1.14E-14	
GO:0007400	neuroblast fate determination	biological process	1.27E-14	
GO:0022603	regulation of anatomical structure morphogenesis	biological process	1.37E-14	
GO:0048608	reproductive structure development	biological process	1.52E-14	

GO:0009987	cellular process	biological process	1.79E-14	
GO:0003006	developmental process involved in reproduction	biological process	2.44E-14	
GO:0008134	transcription factor binding	molecular function	2.46E-14	
GO:0007422	peripheral nervous system development	biological process	5.35E-14	
GO:0031492	nucleosomal DNA binding	molecular function	5.61E-14	
GO:0030030	cell projection organization	biological process	1.39E-13	
GO:0060562	epithelial tube morphogenesis	biological process	1.60E-13	
GO:0042127	regulation of cell population proliferation	biological process	2.64E-13	
GO:0040011	locomotion	biological process	3.59E-13	
GO:0008150	biological_process	biological process	3.77E-13	
GO:0048592	eye morphogenesis	biological process	3.96E-13	
GO:0090596	sensory organ morphogenesis	biological process	3.96E-13	
GO:2000027	regulation of animal organ morphogenesis	biological process	3.96E-13	
GO:0008406	gonad development	biological process	4.21E-13	
GO:0007525	somatic muscle development	biological process	4.87E-13	
GO:0035120	post-embryonic appendage morphogenesis	biological process	6.59E-13	
GO:0043228	non-membrane-bounded organelle	cellular component	8.47E-13	
GO:0043232	intracellular non-membrane-bounded organelle	cellular component	8.47E-13	
GO:0016477	cell migration	biological process	1.14E-12	
GO:0007447	imaginal disc pattern formation	biological process	1.39E-12	
GO:0040012	regulation of locomotion	biological process	1.42E-12	
GO:0051241	negative regulation of multicellular organismal process	biological process	1.53E-12	
GO:0009952	anterior/posterior pattern specification	biological process	1.57E-12	
GO:0051726	regulation of cell cycle	biological process	1.97E-12	
GO:0007476	imaginal disc-derived wing morphogenesis	biological process	2.31E-12	
GO:0043167	ion binding	molecular function		2.94E-12
GO:0007379	segment specification	biological process	3.30E-12	
GO:0042659	regulation of cell fate specification	biological process	3.30E-12	
GO:0048646	anatomical structure formation involved in morphogenesis	biological process	3.89E-12	
GO:0010453	regulation of cell fate commitment	biological process	8.38E-12	
GO:0010160	formation of animal organ boundary	biological process	1.03E-11	
GO:0005700	polytene chromosome	cellular component	1.17E-11	
GO:0007346	regulation of mitotic cell cycle	biological process	1.29E-11	

GO:0008037	cell recognition	biological process	2.42E-11	
GO:0009880	embryonic pattern specification	biological process	2.94E-11	
GO:0008038	neuron recognition	biological process	5.27E-11	
GO:0048870	cell motility	biological process	5.42E-11	
GO:0001216	DNA-binding transcription activator activity	molecular function	5.99E-11	
GO:0110165	cellular anatomical entity	cellular component	6.85E-11	
GO:0035282	segmentation	biological process	8.12E-11	
GO:0048583	regulation of response to stimulus	biological process	9.21E-11	
GO:0046872	metal ion binding	molecular function		1.58E-10
GO:0001228	DNA-binding transcription activator activity, RNA polymerase II-specific	molecular function	2.24E-10	
GO:0007445	determination of imaginal disc primordium	biological process	2.64E-10	
GO:0014019	neuroblast development	biological process	2.64E-10	
GO:0043169	cation binding	molecular function		4.29E-10
GO:0051270	regulation of cellular component movement	biological process	4.88E-10	
GO:0051094	positive regulation of developmental process	biological process	5.65E-10	
GO:0048864	stem cell development	biological process	6.42E-10	
GO:0007560	imaginal disc morphogenesis	biological process	7.67E-10	
GO:0008045	motor neuron axon guidance	biological process	9.49E-10	
GO:0008347	glial cell migration	biological process	1.33E-09	
GO:0048813	dendrite morphogenesis	biological process	1.68E-09	
GO:0001745	compound eye morphogenesis	biological process	1.95E-09	
GO:0007420	brain development	biological process	2.13E-09	
GO:0048563	post-embryonic animal organ morphogenesis	biological process	2.46E-09	
GO:0005667	transcription regulator complex	cellular component	3.18E-09	
GO:0051301	cell division	biological process	3.41E-09	
GO:0035225	determination of genital disc primordium	biological process	3.74E-09	
GO:0001654	eye development	biological process	5.19E-09	
GO:0035287	head segmentation	biological process	5.53E-09	
GO:1903047	mitotic cell cycle process	biological process	5.53E-09	
GO:0050768	negative regulation of neurogenesis	biological process	5.53E-09	
GO:0070983	dendrite guidance	biological process	6.05E-09	
GO:0005575	cellular_component	cellular component	6.71E-09	
GO:0010564	regulation of cell cycle process	biological process	9.39E-09	

GO:0045597	positive regulation of cell differentiation	biological process	9.39E-09	
GO:0046532	regulation of photoreceptor cell differentiation	biological process	1.15E-08	
GO:0007380	specification of segmental identity, head	biological process	1.84E-08	
GO:0007432	salivary gland boundary specification	biological process	1.84E-08	
GO:0110116	regulation of compound eye photoreceptor cell differentiation	biological process	2.51E-08	
GO:0044877	protein-containing complex binding	molecular function	2.65E-08	
GO:0010720	positive regulation of cell development	biological process	3.06E-08	
GO:0031490	chromatin DNA binding	molecular function	3.21E-08	
GO:0051961	negative regulation of nervous system development	biological process	3.48E-08	
GO:0007610	behavior	biological process	3.81E-08	
GO:0016198	axon choice point recognition	biological process	3.89E-08	
GO:0003714	transcription corepressor activity	molecular function	3.94E-08	
GO:0007399	nervous system development	biological process	5.28E-08	
GO:0120035	regulation of plasma membrane bounded cell projection organization	biological process	5.28E-08	
GO:0040008	regulation of growth	biological process	5.38E-08	
GO:0010454	negative regulation of cell fate commitment	biological process	5.90E-08	
GO:0031344	regulation of cell projection organization	biological process	6.11E-08	
GO:0048732	gland development	biological process	7.19E-08	
GO:0098687	chromosomal region	cellular component	7.31E-08	
GO:0010975	regulation of neuron projection development	biological process	7.52E-08	
GO:0048666	neuron development	biological process	8.71E-08	
GO:0042051	compound eye photoreceptor development	biological process	8.94E-08	
GO:0007455	eye-antennal disc morphogenesis	biological process	9.50E-08	
GO:0044786	cell cycle DNA replication	biological process	9.50E-08	
GO:0001667	ameboidal-type cell migration	biological process	1.03E-07	
GO:0007526	larval somatic muscle development	biological process	1.05E-07	
GO:0048749	compound eye development	biological process	1.08E-07	
GO:0010721	negative regulation of cell development	biological process	1.08E-07	
GO:0048569	post-embryonic animal organ development	biological process	1.08E-07	
GO:0048585	negative regulation of response to stimulus	biological process	1.41E-07	
GO:0003712	transcription coregulator activity	molecular function	1.41E-07	
GO:0001227	DNA-binding transcription repressor activity, RNA polymerase II-specific	molecular function	1.47E-07	
GO:0030334	regulation of cell migration	biological process	1.63E-07	

GO:0035222	wing disc pattern formation	biological process	1.76E-07	
GO:0001217	DNA-binding transcription repressor activity	molecular function	1.85E-07	
GO:0042393	histone binding	molecular function	1.85E-07	
GO:0042803	protein homodimerization activity	molecular function	1.85E-07	
GO:0050839	cell adhesion molecule binding	molecular function	2.21E-07	
GO:0040017	positive regulation of locomotion	biological process	2.23E-07	
GO:0035277	spiracle morphogenesis, open tracheal system	biological process	2.26E-07	
GO:0051240	positive regulation of multicellular organismal process	biological process	2.60E-07	
GO:0007169	transmembrane receptor protein tyrosine kinase signaling pathway	biological process	2.68E-07	
GO:0042462	eye photoreceptor cell development	biological process	2.75E-07	
GO:0043549	regulation of kinase activity	biological process	3.10E-07	
GO:0007444	imaginal disc development	biological process	3.16E-07	
GO:0045665	negative regulation of neuron differentiation	biological process	3.61E-07	
GO:2000145	regulation of cell motility	biological process	3.80E-07	
GO:0008283	cell population proliferation	biological process	4.73E-07	
GO:0009996	negative regulation of cell fate specification	biological process	4.73E-07	
GO:0051338	regulation of transferase activity	biological process	4.84E-07	
GO:0042023	DNA endoreduplication	biological process	5.07E-07	
GO:0007507	heart development	biological process	5.44E-07	
GO:0007517	muscle organ development	biological process	5.44E-07	
GO:0000785	chromatin	cellular component	6.28E-07	
GO:0008356	asymmetric cell division	biological process	6.98E-07	
GO:0035289	posterior head segmentation	biological process	7.12E-07	
GO:0050673	epithelial cell proliferation	biological process	7.12E-07	
GO:0061331	epithelial cell proliferation involved in Malpighian tubule morphogenesis	biological process	7.12E-07	
GO:2001013	epithelial cell proliferation involved in renal tubule morphogenesis	biological process	7.12E-07	
GO:0016318	ommatidial rotation	biological process	7.41E-07	
GO:2000177	regulation of neural precursor cell proliferation	biological process	7.72E-07	
GO:0008284	positive regulation of cell population proliferation	biological process	7.93E-07	
GO:0010631	epithelial cell migration	biological process	8.86E-07	
GO:0042461	photoreceptor cell development	biological process	9.17E-07	
GO:0007059	chromosome segregation	biological process	1.00E-06	
GO:0007409	axonogenesis	biological process	1.00E-06	

GO:0009790	embryo development	biological process	1.01E-06	
GO:0009792	embryo development ending in birth or egg hatching	biological process	1.01E-06	
GO:0098813	nuclear chromosome segregation	biological process	1.01E-06	
GO:0046533	negative regulation of photoreceptor cell differentiation	biological process	1.02E-06	
GO:0048638	regulation of developmental growth	biological process	1.11E-06	
GO:0007424	open tracheal system development	biological process	1.16E-06	
GO:0060541	respiratory system development	biological process	1.16E-06	
GO:0032991	protein-containing complex	cellular component	1.57E-06	
GO:0007435	salivary gland morphogenesis	biological process	1.63E-06	
GO:0022612	gland morphogenesis	biological process	1.63E-06	
GO:0090175	regulation of establishment of planar polarity	biological process	1.73E-06	
GO:0009953	dorsal/ventral pattern formation	biological process	1.73E-06	
GO:0035051	cardiocyte differentiation	biological process	1.86E-06	
GO:0016199	axon midline choice point recognition	biological process	1.92E-06	
GO:0045926	negative regulation of growth	biological process	1.98E-06	
GO:0007498	mesoderm development	biological process	2.03E-06	
GO:0048477	oogenesis	biological process	2.10E-06	
GO:0008270	zinc ion binding	molecular function		2.16E-06
GO:0007292	female gamete generation	biological process	2.37E-06	
GO:0007494	midgut development	biological process	2.60E-06	
GO:0007450	dorsal/ventral pattern formation, imaginal disc	biological process	2.66E-06	
GO:0098609	cell-cell adhesion	biological process	2.74E-06	
GO:0017053	transcription repressor complex	cellular component	2.76E-06	
GO:0030155	regulation of cell adhesion	biological process	3.47E-06	
GO:0009966	regulation of signal transduction	biological process	3.58E-06	
GO:0007155	cell adhesion	biological process	3.64E-06	
GO:0007156	homophilic cell adhesion via plasma membrane adhesion molecules	biological process	3.77E-06	
GO:0050769	positive regulation of neurogenesis	biological process	4.07E-06	
GO:0006333	chromatin assembly or disassembly	biological process	4.09E-06	
GO:0061318	renal filtration cell differentiation	biological process	4.26E-06	
GO:0061319	nephrocyte differentiation	biological process	4.26E-06	
GO:0051962	positive regulation of nervous system development	biological process	5.22E-06	
GO:0006935	chemotaxis	biological process	5.52E-06	

GO:0022407	regulation of cell-cell adhesion	biological process	5.52E-06	
GO:0050920	regulation of chemotaxis	biological process	5.52E-06	
GO:0016331	morphogenesis of embryonic epithelium	biological process	6.73E-06	
GO:0051272	positive regulation of cellular component movement	biological process	7.64E-06	
GO:0042802	identical protein binding	molecular function	7.82E-06	
GO:0030335	positive regulation of cell migration	biological process	9.19E-06	
GO:0022610	biological adhesion	biological process	9.44E-06	
GO:0046530	photoreceptor cell differentiation	biological process	9.44E-06	
GO:0009798	axis specification	biological process	9.75E-06	
GO:1905330	regulation of morphogenesis of an epithelium	biological process	1.07E-05	
GO:0009948	anterior/posterior axis specification	biological process	1.21E-05	
GO:0045611	negative regulation of hemocyte differentiation	biological process	1.29E-05	
GO:0001558	regulation of cell growth	biological process	1.34E-05	
GO:0009968	negative regulation of signal transduction	biological process	1.41E-05	
GO:0006338	chromatin remodeling	biological process	1.52E-05	
GO:0048565	digestive tract development	biological process	1.57E-05	
GO:0005654	nucleoplasm	cellular component	1.61E-05	
GO:0034329	cell junction assembly	biological process	1.74E-05	
GO:0045467	R7 cell development	biological process	1.78E-05	
GO:0007469	antennal development	biological process	1.80E-05	
GO:0010001	glial cell differentiation	biological process	1.80E-05	
GO:0045466	R7 cell differentiation	biological process	1.80E-05	
GO:0110118	negative regulation of compound eye photoreceptor cell differentiation	biological process	1.80E-05	
GO:0140097	catalytic activity, acting on DNA	molecular function		1.83E-05
GO:0098742	cell-cell adhesion via plasma-membrane adhesion molecules	biological process	1.90E-05	
GO:0045610	regulation of hemocyte differentiation	biological process	1.94E-05	
GO:0010648	negative regulation of cell communication	biological process	1.99E-05	
GO:0070161	anchoring junction	cellular component	2.04E-05	
GO:0023057	negative regulation of signaling	biological process	2.07E-05	
GO:0061326	renal tubule development	biological process	2.09E-05	
GO:0072002	Malpighian tubule development	biological process	2.09E-05	
GO:2000147	positive regulation of cell motility	biological process	2.14E-05	
GO:0007391	dorsal closure	biological process	2.41E-05	

GO:0010769	regulation of cell morphogenesis involved in differentiation	biological process	2.41E-05	
GO:0010632	regulation of epithelial cell migration	biological process	2.48E-05	
GO:0006468	protein phosphorylation	biological process		2.83E-05
GO:0022604	regulation of cell morphogenesis	biological process	2.91E-05	
GO:0048190	wing disc dorsal/ventral pattern formation	biological process	3.18E-05	
GO:1901987	regulation of cell cycle phase transition	biological process	3.32E-05	
GO:0035050	embryonic heart tube development	biological process	3.35E-05	
GO:0005524	ATP binding	molecular function		3.35E-05
GO:0051347	positive regulation of transferase activity	biological process	3.70E-05	
GO:0050770	regulation of axonogenesis	biological process	3.97E-05	
GO:0072499	photoreceptor cell axon guidance	biological process	4.21E-05	
GO:0004672	protein kinase activity	molecular function		4.40E-05
GO:0003678	DNA helicase activity	molecular function		4.56E-05
GO:0003730	mRNA 3'-UTR binding	molecular function	4.92E-05	
GO:0007474	imaginal disc-derived wing vein specification	biological process	5.09E-05	
GO:0008407	chaeta morphogenesis	biological process	5.09E-05	
GO:0006277	DNA amplification	biological process	5.24E-05	
GO:0032559	adenyl ribonucleotide binding	molecular function		5.75E-05
GO:0017145	stem cell division	biological process	5.77E-05	
GO:0001700	embryonic development via the syncytial blastoderm	biological process	5.85E-05	
GO:0030554	adenyl nucleotide binding	molecular function		5.86E-05
GO:0034330	cell junction organization	biological process	6.03E-05	
GO:0033674	positive regulation of kinase activity	biological process	6.75E-05	
GO:0032879	regulation of localization	biological process	6.78E-05	
GO:0045859	regulation of protein kinase activity	biological process	6.87E-05	
GO:0002682	regulation of immune system process	biological process	7.36E-05	
GO:0035153	epithelial cell type specification, open tracheal system	biological process	7.36E-05	
GO:0007281	germ cell development	biological process	7.83E-05	
GO:0009954	proximal/distal pattern formation	biological process	7.87E-05	
GO:1901990	regulation of mitotic cell cycle phase transition	biological process	7.89E-05	
GO:0045448	mitotic cell cycle, embryonic	biological process	8.49E-05	
GO:0001751	compound eye photoreceptor cell differentiation	biological process	8.56E-05	
GO:0045931	positive regulation of mitotic cell cycle	biological process	8.56E-05	

GO:1904949	ATPase complex	cellular component	8.96E-05	
GO:0007297	ovarian follicle cell migration	biological process	9.23E-05	
GO:0035288	anterior head segmentation	biological process	9.60E-05	
GO:0051890	regulation of cardioblast differentiation	biological process	9.60E-05	
GO:0060563	neuroepithelial cell differentiation	biological process	9.60E-05	
GO:0061101	neuroendocrine cell differentiation	biological process	9.60E-05	
GO:0070603	SWI/SNF superfamily-type complex	cellular component	9.61E-05	
GO:0048534	hematopoietic or lymphoid organ development	biological process	9.97E-05	
GO:0021782	glial cell development	biological process	0.0001032104796	
GO:0008094	DNA-dependent ATPase activity	molecular function		0.0001036942719
GO:0032040	small-subunit processome	cellular component	0.0001080049527	
GO:0040007	growth	biological process	0.0001080510832	
GO:0048589	developmental growth	biological process	0.0001080510832	
GO:0019904	protein domain specific binding	molecular function	0.000110373254	
GO:0045676	regulation of R7 cell differentiation	biological process	0.0001136890076	
GO:0072091	regulation of stem cell proliferation	biological process	0.0001160475399	
GO:0045787	positive regulation of cell cycle	biological process	0.0001166486259	
GO:0048640	negative regulation of developmental growth	biological process	0.0001166486259	
GO:0000278	mitotic cell cycle	biological process	0.0001201088695	
GO:0045746	negative regulation of Notch signaling pathway	biological process	0.0001350228394	
GO:0017148	negative regulation of translation	biological process	0.0001414556701	
GO:0140297	DNA-binding transcription factor binding	molecular function	0.0001546851924	
GO:0000070	mitotic sister chromatid segregation	biological process	0.0001570517849	
GO:0110111	negative regulation of animal organ morphogenesis	biological process	0.0001570517849	
GO:1902667	regulation of axon guidance	biological process	0.0001570517849	
GO:0007276	gamete generation	biological process	0.0001592014144	
GO:0048865	stem cell fate commitment	biological process	0.0001592014144	
GO:0034249	negative regulation of cellular amide metabolic process	biological process	0.00017082274	
GO:0001754	eye photoreceptor cell differentiation	biological process	0.0001743122525	
GO:1903311	regulation of mRNA metabolic process	biological process	0.0001769394859	
GO:0007298	border follicle cell migration	biological process	0.0001801376904	
GO:0042330	taxis	biological process	0.0001801376904	
GO:0007611	learning or memory	biological process	0.0001826960645	

GO:0010646	regulation of cell communication	biological process	0.0001844917594	
GO:0023051	regulation of signaling	biological process	0.0001844917594	
GO:0048663	neuron fate commitment	biological process	0.0001956862907	
GO:0050890	cognition	biological process	0.0002013519762	
GO:0007307	eggshell chorion gene amplification	biological process	0.0002032941725	
GO:0033301	cell cycle comprising mitosis without cytokinesis	biological process	0.0002032941725	
GO:0035146	tube fusion	biological process	0.0002032941725	
GO:0035147	branch fusion, open tracheal system	biological process	0.0002032941725	
GO:0043067	regulation of programmed cell death	biological process	0.0002032941725	
GO:0050684	regulation of mRNA processing	biological process	0.0002032941725	
GO:0000819	sister chromatid segregation	biological process	0.0002034776264	
GO:0055057	neuroblast division	biological process	0.0002034776264	
GO:0007049	cell cycle	biological process	0.0002054443601	
GO:0000727	double-strand break repair via break-induced replication	biological process	0.000216931291	
GO:0007522	visceral muscle development	biological process	0.000216931291	
GO:0048736	appendage development	biological process	0.000216931291	
GO:0006275	regulation of DNA replication	biological process	0.0002176545378	
GO:0048542	lymph gland development	biological process	0.0002192528104	
GO:1903046	meiotic cell cycle process	biological process	0.0002621971606	
GO:0043068	positive regulation of programmed cell death	biological process	0.0002662476291	
GO:2000736	regulation of stem cell differentiation	biological process	0.0002699848418	
GO:0007398	ectoderm development	biological process	0.0002772746886	
GO:1905207	regulation of cardiocyte differentiation	biological process	0.0002772746886	
GO:0007350	blastoderm segmentation	biological process	0.0002999612611	
GO:0007448	anterior/posterior pattern specification, imaginal disc	biological process	0.0003059928332	
GO:0046552	photoreceptor cell fate commitment	biological process	0.0003284003083	
GO:1902692	regulation of neuroblast proliferation	biological process	0.0003284003083	
GO:0030371	translation repressor activity	molecular function	0.0003299854227	
GO:0033043	regulation of organelle organization	biological process	0.0003471196508	
GO:0007416	synapse assembly	biological process	0.0003475628038	
GO:0090659	walking behavior	biological process	0.0003527236486	
GO:0008039	synaptic target recognition	biological process	0.0003530040092	
GO:0048609	multicellular organismal reproductive process	biological process	0.0003559047055	

GO:0006261	DNA-dependent DNA replication	biological process	0.0004043806489	
GO:0007365	periodic partitioning	biological process	0.0004043806489	
GO:0046425	regulation of receptor signaling pathway via JAK-STAT	biological process	0.0004154291025	
GO:1904892	regulation of receptor signaling pathway via STAT	biological process	0.0004154291025	
GO:0016360	sensory organ precursor cell fate determination	biological process	0.0004300607968	
GO:0050919	negative chemotaxis	biological process	0.0004300607968	
GO:0060582	cell fate determination involved in pattern specification	biological process	0.0004300607968	
GO:0051129	negative regulation of cellular component organization	biological process	0.0004498481649	
GO:0046620	regulation of organ growth	biological process	0.0004835045618	
GO:0044260	cellular macromolecule metabolic process	biological process		0.0004900966471
GO:0048024	regulation of mRNA splicing, via spliceosome	biological process	0.0005357307388	
GO:0002065	columnar/cuboidal epithelial cell differentiation	biological process	0.0005464422865	
GO:0007442	hindgut morphogenesis	biological process	0.0005464422865	
GO:0035186	syncytial blastoderm mitotic cell cycle	biological process	0.0005464422865	
GO:1990709	presynaptic active zone organization	biological process	0.0005464422865	
GO:0031290	retinal ganglion cell axon guidance	biological process	0.0005465772334	
GO:0048584	positive regulation of response to stimulus	biological process	0.0005465772334	
GO:0030516	regulation of axon extension	biological process	0.0005494217494	
GO:0055059	asymmetric neuroblast division	biological process	0.0005494217494	
GO:0061387	regulation of extent of cell growth	biological process	0.0005494217494	
GO:0090090	negative regulation of canonical Wnt signaling pathway	biological process	0.0005494217494	
GO:0008595	anterior/posterior axis specification, embryo	biological process	0.0006040051636	
GO:0001704	formation of primary germ layer	biological process	0.000642279603	
GO:0007438	oenocyte development	biological process	0.000642279603	
GO:0007440	foregut morphogenesis	biological process	0.000642279603	
GO:0035221	genital disc pattern formation	biological process	0.000642279603	
GO:0035224	genital disc anterior/posterior pattern formation	biological process	0.000642279603	
GO:0060573	cell fate specification involved in pattern specification	biological process	0.000642279603	
GO:0071162	CMG complex	cellular component	0.0006654061887	
GO:0035639	purine ribonucleoside triphosphate binding	molecular function		0.0006732344485
GO:0002683	negative regulation of immune system process	biological process	0.0006945581059	
GO:0098722	asymmetric stem cell division	biological process	0.0007181005894	
GO:0010941	regulation of cell death	biological process	0.0007662840611	

GO:0016310	phosphorylation	biological process		0.0008072839397
GO:0030534	adult behavior	biological process	0.0008155995942	
GO:0008354	germ cell migration	biological process	0.0008389604499	
GO:0030178	negative regulation of Wnt signaling pathway	biological process	0.0008389604499	
GO:0005911	cell-cell junction	cellular component	0.0008711933938	
GO:0016348	imaginal disc-derived leg joint morphogenesis	biological process	0.0009030097511	
GO:0042387	plasmatocyte differentiation	biological process	0.0009030097511	
GO:0042478	regulation of eye photoreceptor cell development	biological process	0.0009030097511	
GO:0042689	regulation of crystal cell differentiation	biological process	0.0009030097511	
GO:0045613	regulation of plasmatocyte differentiation	biological process	0.0009030097511	
GO:0048806	genitalia development	biological process	0.0009030097511	
GO:0048665	neuron fate specification	biological process	0.0009361093106	
GO:0050808	synapse organization	biological process	0.000969624178	
GO:0043902	positive regulation of multi-organism process	biological process	0.0009729477613	
GO:0001742	oenocyte differentiation	biological process	0.001006730796	
GO:0001752	compound eye photoreceptor fate commitment	biological process	0.001006730796	
GO:0007521	muscle cell fate determination	biological process	0.001006730796	
GO:0016200	synaptic target attraction	biological process	0.001006730796	
GO:0035284	brain segmentation	biological process	0.001006730796	
GO:0036058	filtration diaphragm assembly	biological process	0.001006730796	
GO:0042686	regulation of cardioblast cell fate specification	biological process	0.001006730796	
GO:0042706	eye photoreceptor cell fate commitment	biological process	0.001006730796	
GO:2000043	regulation of cardiac cell fate specification	biological process	0.001006730796	
GO:0032555	purine ribonucleotide binding	molecular function		0.001022908113
GO:0005730	nucleolus	cellular component	0.001090220333	
GO:0032508	DNA duplex unwinding	biological process	0.001096707955	
GO:0042386	hemocyte differentiation	biological process	0.001096707955	
GO:0042325	regulation of phosphorylation	biological process	0.001123437788	
GO:0048863	stem cell differentiation	biological process	0.001141923341	
GO:0031261	DNA replication preinitiation complex	cellular component	0.001145683225	
GO:0030684	preribosome	cellular component	0.001156532413	
GO:0030424	axon	cellular component	0.001179953338	
GO:0000904	cell morphogenesis involved in differentiation	biological process	0.001214064585	

GO:0007418	ventral midline development	biological process	0.001253804027	
GO:0007523	larval visceral muscle development	biological process	0.001253804027	
GO:0046667	compound eye retinal cell programmed cell death	biological process	0.001253804027	
GO:0048664	neuron fate determination	biological process	0.001253804027	
GO:0061382	Malpighian tubule tip cell differentiation	biological process	0.001253804027	
GO:0017076	purine nucleotide binding	molecular function		0.001282386012
GO:0046914	transition metal ion binding	molecular function		0.001282386012
GO:0007613	memory	biological process	0.001283427402	
GO:0032268	regulation of cellular protein metabolic process	biological process	0.001368185127	
GO:0035206	regulation of hemocyte proliferation	biological process	0.001392247863	
GO:0051246	regulation of protein metabolic process	biological process	0.001400433858	
GO:0042981	regulation of apoptotic process	biological process	0.001400882356	
GO:0070192	chromosome organization involved in meiotic cell cycle	biological process	0.001409898101	
GO:0007173	epidermal growth factor receptor signaling pathway	biological process	0.001444436731	
GO:0038127	ERBB signaling pathway	biological process	0.001444436731	
GO:0035220	wing disc development	biological process	0.001523022142	
GO:0007616	long-term memory	biological process	0.001555395204	
GO:0035223	leg disc pattern formation	biological process	0.001596462511	
GO:0051783	regulation of nuclear division	biological process	0.001749784277	
GO:0017116	single-stranded DNA helicase activity	molecular function	0.001781340909	
GO:0016319	mushroom body development	biological process	0.001794751184	
GO:0060828	regulation of canonical Wnt signaling pathway	biological process	0.001869838126	
GO:0032553	ribonucleotide binding	molecular function		0.001892599305
GO:0000578	embryonic axis specification	biological process	0.001898073155	
GO:0032392	DNA geometric change	biological process	0.001898073155	
GO:0007088	regulation of mitotic nuclear division	biological process	0.002051959805	
GO:0030858	positive regulation of epithelial cell differentiation	biological process	0.002070990573	
GO:0071897	DNA biosynthetic process	biological process	0.002070990573	
GO:0007628	adult walking behavior	biological process	0.002117083623	
GO:0022409	positive regulation of cell-cell adhesion	biological process	0.002117083623	
GO:0031399	regulation of protein modification process	biological process	0.002133649693	
GO:0032101	regulation of response to external stimulus	biological process	0.002184243751	
GO:0007431	salivary gland development	biological process	0.002189155517	

GO:0000381	regulation of alternative mRNA splicing, via spliceosome	biological process	0.002206515478	
GO:0007096	regulation of exit from mitosis	biological process	0.002206515478	
GO:0046847	filopodium assembly	biological process	0.002206515478	
GO:0048036	central complex development	biological process	0.002206515478	
GO:0061320	pericardial nephrocyte differentiation	biological process	0.002206515478	
GO:0000808	origin recognition complex	cellular component		0.002428261182
GO:2000648	positive regulation of stem cell proliferation	biological process	0.002438933002	
GO:0000792	heterochromatin	cellular component	0.002566351905	
GO:0008052	sensory organ boundary specification	biological process	0.002571983621	
GO:0043388	positive regulation of DNA binding	biological process	0.002571983621	
GO:0045317	equator specification	biological process	0.002571983621	
GO:1900087	positive regulation of G1/S transition of mitotic cell cycle	biological process	0.002571983621	
GO:1902808	positive regulation of cell cycle G1/S phase transition	biological process	0.002571983621	
GO:0043484	regulation of RNA splicing	biological process	0.002632312117	
GO:0045170	spectrosome	cellular component	0.002633623405	
GO:0008593	regulation of Notch signaling pathway	biological process	0.00267362016	
GO:0003729	mRNA binding	molecular function	0.002844781628	
GO:0007480	imaginal disc-derived leg morphogenesis	biological process	0.002867649007	
GO:1902806	regulation of cell cycle G1/S phase transition	biological process	0.002933530186	
GO:2000045	regulation of G1/S transition of mitotic cell cycle	biological process	0.002933530186	
GO:0048754	branching morphogenesis of an epithelial tube	biological process	0.002970821394	
GO:1902275	regulation of chromatin organization	biological process	0.002970821394	
GO:0007479	leg disc proximal/distal pattern formation	biological process	0.003042536642	
GO:0008156	negative regulation of DNA replication	biological process	0.003042536642	
GO:0042688	crystal cell differentiation	biological process	0.003042536642	
GO:0055001	muscle cell development	biological process	0.003042536642	
GO:0007403	glial cell fate determination	biological process	0.003087359347	
GO:0007429	secondary branching, open tracheal system	biological process	0.003087359347	
GO:0014018	neuroblast fate specification	biological process	0.003087359347	
GO:0035385	Roundabout signaling pathway	biological process	0.003087359347	
GO:0048866	stem cell fate specification	biological process	0.003087359347	
GO:0045786	negative regulation of cell cycle	biological process	0.003202532835	
GO:0090068	positive regulation of cell cycle process	biological process	0.003329150001	

GO:0008258	head involution	biological process	0.003482044524	
GO:0001763	morphogenesis of a branching structure	biological process	0.003508099714	
GO:0030856	regulation of epithelial cell differentiation	biological process	0.003508099714	
GO:0061138	morphogenesis of a branching epithelium	biological process	0.003508099714	
GO:0006268	DNA unwinding involved in DNA replication	biological process	0.003571966652	
GO:0007482	haltere development	biological process	0.003571966652	
GO:0045614	negative regulation of plasmatocyte differentiation	biological process	0.003571966652	
GO:0046666	retinal cell programmed cell death	biological process	0.003571966652	
GO:0048790	maintenance of presynaptic active zone structure	biological process	0.003571966652	
GO:0051304	chromosome separation	biological process	0.003571966652	
GO:0016773	phosphotransferase activity, alcohol group as acceptor	molecular function		0.003771655533
GO:0010942	positive regulation of cell death	biological process	0.00390816122	
GO:0030054	cell junction	cellular component	0.004315303232	
GO:0006996	organelle organization	biological process	0.004408089672	
GO:0007626	locomotory behavior	biological process	0.004408089672	
GO:0019827	stem cell population maintenance	biological process	0.004408089672	
GO:0098727	maintenance of cell number	biological process	0.004408089672	
GO:0060249	anatomical structure homeostasis	biological process	0.004532320564	
GO:0033044	regulation of chromosome organization	biological process	0.004798121153	
GO:0007443	Malpighian tubule morphogenesis	biological process	0.004816495361	
GO:0035332	positive regulation of hippo signaling	biological process	0.004816495361	
GO:0051098	regulation of binding	biological process	0.004816495361	
GO:0061333	renal tubule morphogenesis	biological process	0.004816495361	
GO:1905881	positive regulation of oogenesis	biological process	0.00493582979	
GO:0030308	negative regulation of cell growth	biological process	0.004990974738	
GO:1903688	positive regulation of border follicle cell migration	biological process	0.005098032446	
GO:0007402	ganglion mother cell fate determination	biological process	0.005151046699	
GO:0010032	meiotic chromosome condensation	biological process	0.005151046699	
GO:0016204	determination of muscle attachment site	biological process	0.005151046699	
GO:0042690	negative regulation of crystal cell differentiation	biological process	0.005151046699	
GO:0042694	muscle cell fate specification	biological process	0.005151046699	
GO:0045677	negative regulation of R7 cell differentiation	biological process	0.005151046699	
GO:0050918	positive chemotaxis	biological process	0.005151046699	

GO:0003697	single-stranded DNA binding	molecular function	0.005195392504	
GO:0030111	regulation of Wnt signaling pathway	biological process	0.005333783157	
GO:0007464	R3/R4 cell fate commitment	biological process	0.005543658215	
GO:0045314	regulation of compound eye photoreceptor development	biological process	0.005543658215	
GO:0061327	anterior Malpighian tubule development	biological process	0.005543658215	
GO:0030707	ovarian follicle cell development	biological process	0.005677474335	
GO:0000079	regulation of cyclin-dependent protein serine/threonine kinase activity	biological process	0.00580231283	
GO:1904029	regulation of cyclin-dependent protein kinase activity	biological process	0.00580231283	
GO:2000243	positive regulation of reproductive process	biological process	0.005921339723	
GO:0007367	segment polarity determination	biological process	0.006016884465	
GO:0010634	positive regulation of epithelial cell migration	biological process	0.006016884465	
GO:0003727	single-stranded RNA binding	molecular function	0.00607142362	
GO:0005886	plasma membrane	cellular component	0.006077462692	
GO:0008543	fibroblast growth factor receptor signaling pathway	biological process	0.006281253143	
GO:0046621	negative regulation of organ growth	biological process	0.006281253143	
GO:0110110	positive regulation of animal organ morphogenesis	biological process	0.006281253143	
GO:2000179	positive regulation of neural precursor cell proliferation	biological process	0.006281253143	
GO:0002066	columnar/cuboidal epithelial cell development	biological process	0.006337886134	
GO:0045666	positive regulation of neuron differentiation	biological process	0.006556962541	
GO:0044295	axonal growth cone	cellular component	0.006638291024	
GO:0050773	regulation of dendrite development	biological process	0.006657289381	
GO:0043065	positive regulation of apoptotic process	biological process	0.006689779942	
GO:0090575	RNA polymerase II transcription regulator complex	cellular component	0.00689638962	
GO:1903684	regulation of border follicle cell migration	biological process	0.007158415353	
GO:0006310	DNA recombination	biological process	0.007321435466	
GO:0004386	helicase activity	molecular function		0.007492973556
GO:0043138	3'-5' DNA helicase activity	molecular function	0.007612351397	
GO:0007162	negative regulation of cell adhesion	biological process	0.007809118689	
GO:0007449	proximal/distal pattern formation, imaginal disc	biological process	0.007809118689	
GO:0000166	nucleotide binding	molecular function		0.007827630665
GO:1901265	nucleoside phosphate binding	molecular function		0.007827630665
GO:0090329	regulation of DNA-dependent DNA replication	biological process	0.007992994214	
GO:0006302	double-strand break repair	biological process	0.008161462205	

GO:0048737	imaginal disc-derived appendage development	biological process	0.008161462205	
GO:0099558	maintenance of synapse structure	biological process	0.008161462205	
GO:0016301	kinase activity	molecular function		0.008225649685
GO:0005856	cytoskeleton	cellular component	0.008382300819	
GO:0031056	regulation of histone modification	biological process	0.00847823745	
GO:0045570	regulation of imaginal disc growth	biological process	0.00847823745	
GO:0060446	branching involved in open tracheal system development	biological process	0.00847823745	
GO:0031497	chromatin assembly	biological process	0.008613323406	
GO:0016203	muscle attachment	biological process	0.008667537074	
GO:0030855	epithelial cell differentiation	biological process	0.008667537074	
GO:0000724	double-strand break repair via homologous recombination	biological process	0.008979020151	
GO:0000725	recombinational repair	biological process	0.008979020151	
GO:0008584	male gonad development	biological process	0.009111596668	
GO:0010002	cardioblast differentiation	biological process	0.009111596668	
GO:0051383	kinetochore organization	biological process	0.009111596668	
GO:0006464	cellular protein modification process	biological process		0.009560459623
GO:0036211	protein modification process	biological process		0.009560459623
GO:0001932	regulation of protein phosphorylation	biological process	0.00994803946	
GO:0001706	endoderm formation	biological process	0.009971542696	
GO:0002064	epithelial cell development	biological process	0.009971542696	
GO:0003151	outflow tract morphogenesis	biological process	0.009971542696	
GO:0009997	negative regulation of cardioblast cell fate specification	biological process	0.009971542696	
GO:0010092	specification of animal organ identity	biological process	0.009971542696	
GO:0035154	terminal cell fate specification, open tracheal system	biological process	0.009971542696	
GO:0035561	regulation of chromatin binding	biological process	0.009971542696	
GO:0036059	nephrocyte diaphragm assembly	biological process	0.009971542696	
GO:0042063	gliogenesis	biological process	0.009971542696	
GO:0044728	DNA methylation or demethylation	biological process	0.009971542696	
GO:0051382	kinetochore assembly	biological process	0.009971542696	
GO:0051892	negative regulation of cardioblast differentiation	biological process	0.009971542696	
GO:0061321	garland nephrocyte differentiation	biological process	0.009971542696	
GO:1905208	negative regulation of cardiocyte differentiation	biological process	0.009971542696	
GO:2000044	negative regulation of cardiac cell fate specification	biological process	0.009971542696	

GO:0051303	establishment of chromosome localization	biological process	0.01003184937	
GO:0030261	chromosome condensation	biological process	0.01020438457	
GO:0007427	epithelial cell migration, open tracheal system	biological process	0.01024540071	
GO:0000775	chromosome, centromeric region	cellular component	0.01073855285	
GO:0043412	macromolecule modification	biological process		0.01081352252
GO:0022408	negative regulation of cell-cell adhesion	biological process	0.0113442078	
GO:0035065	regulation of histone acetylation	biological process	0.0113442078	
GO:0048634	regulation of muscle organ development	biological process	0.0113442078	
GO:0048800	antennal morphogenesis	biological process	0.0113442078	
GO:1901983	regulation of protein acetylation	biological process	0.0113442078	
GO:2000756	regulation of peptidyl-lysine acetylation	biological process	0.0113442078	
GO:0001894	tissue homeostasis	biological process	0.01136968005	
GO:0040014	regulation of multicellular organism growth	biological process	0.01136968005	
GO:0042058	regulation of epidermal growth factor receptor signaling pathway	biological process	0.01136968005	
GO:1901184	regulation of ERBB signaling pathway	biological process	0.01136968005	
GO:0009967	positive regulation of signal transduction	biological process	0.0114537456	
GO:0048495	Roundabout binding	molecular function	0.01162553656	
GO:0005701	polytene chromosome chromocenter	cellular component	0.01166310223	
GO:0032269	negative regulation of cellular protein metabolic process	biological process	0.01174828323	
GO:0032200	telomere organization	biological process	0.0121830199	
GO:0051248	negative regulation of protein metabolic process	biological process	0.0121830199	
GO:0010647	positive regulation of cell communication	biological process	0.0125359341	
GO:0023056	positive regulation of signaling	biological process	0.0125359341	
GO:0031507	heterochromatin assembly	biological process	0.01280052257	
GO:0034401	chromatin organization involved in regulation of transcription	biological process	0.01280052257	
GO:0097549	chromatin organization involved in negative regulation of transcription	biological process	0.01280052257	
GO:0007413	axonal fasciculation	biological process	0.01284597431	
GO:0010369	chromocenter	cellular component	0.01322280993	
GO:0008586	imaginal disc-derived wing vein morphogenesis	biological process	0.0132467487	
GO:0031401	positive regulation of protein modification process	biological process	0.01329314102	
GO:0031175	neuron projection development	biological process	0.01330839446	
GO:0006352	DNA-templated transcription, initiation	biological process		0.01437415859
GO:0001746	Bolwig's organ morphogenesis	biological process	0.01444734301	

GO:0048568	embryonic organ development	biological process	0.01444734301	
GO:0051099	positive regulation of binding	biological process	0.01444734301	
GO:0060571	morphogenesis of an epithelial fold	biological process	0.01444734301	
GO:0034332	adherens junction organization	biological process	0.01463046171	
GO:0016278	lysine N-methyltransferase activity	molecular function		0.01465036707
GO:0016279	protein-lysine N-methyltransferase activity	molecular function		0.01465036707
GO:0018024	histone-lysine N-methyltransferase activity	molecular function		0.01465036707
GO:0042054	histone methyltransferase activity	molecular function		0.01465036707
GO:0030307	positive regulation of cell growth	biological process	0.01535544212	
GO:0016201	synaptic target inhibition	biological process	0.01547366251	
GO:0035168	larval lymph gland hemocyte differentiation	biological process	0.01547366251	
GO:0045478	fusome organization	biological process	0.01547366251	
GO:0010171	body morphogenesis	biological process	0.01632798533	
GO:0034331	cell junction maintenance	biological process	0.01632798533	
GO:0106030	neuron projection fasciculation	biological process	0.01632798533	
GO:1905879	regulation of oogenesis	biological process	0.01656522912	
GO:0005704	polytene chromosome band	cellular component	0.01672933636	
GO:0040029	regulation of gene expression, epigenetic	biological process	0.01700412717	
GO:0071900	regulation of protein serine/threonine kinase activity	biological process	0.01700412717	
GO:0042327	positive regulation of phosphorylation	biological process	0.01756706075	
GO:0019220	regulation of phosphate metabolic process	biological process	0.01808997297	
GO:0051174	regulation of phosphorus metabolic process	biological process	0.01808997297	
GO:0010948	negative regulation of cell cycle process	biological process	0.01822950411	
GO:0045930	negative regulation of mitotic cell cycle	biological process	0.01822950411	
GO:0045216	cell-cell junction organization	biological process	0.0184122945	
GO:0065009	regulation of molecular function	biological process	0.01882330958	
GO:0097367	carbohydrate derivative binding	molecular function		0.01901510514
GO:0005604	basement membrane	cellular component	0.01965410402	
GO:0005917	nephrocyte diaphragm	cellular component	0.01965410402	
GO:0031213	RSF complex	cellular component	0.01965410402	
GO:0036056	filtration diaphragm	cellular component	0.01965410402	
GO:0051302	regulation of cell division	biological process	0.02049351603	
GO:0050000	chromosome localization	biological process	0.02062147588	

GO:0003013	circulatory system process	biological process	0.02087679759	
GO:0003015	heart process	biological process	0.02087679759	
GO:0006304	DNA modification	biological process	0.02135353956	
GO:0007501	mesodermal cell fate specification	biological process	0.02135353956	
GO:0007502	digestive tract mesoderm development	biological process	0.02135353956	
GO:0007540	sex determination, establishment of X:A ratio	biological process	0.02135353956	
GO:0014017	neuroblast fate commitment	biological process	0.02135353956	
GO:0031509	subtelomeric heterochromatin assembly	biological process	0.02135353956	
GO:0032202	telomere assembly	biological process	0.02135353956	
GO:0035171	lamellocyte differentiation	biological process	0.02135353956	
GO:0035310	notum cell fate specification	biological process	0.02135353956	
GO:0042682	regulation of compound eye cone cell fate specification	biological process	0.02135353956	
GO:0060323	head morphogenesis	biological process	0.02135353956	
GO:0060911	cardiac cell fate commitment	biological process	0.02135353956	
GO:0071168	protein localization to chromatin	biological process	0.02135353956	
GO:0140461	subtelomeric heterochromatin organization	biological process	0.02135353956	
GO:0035202	tracheal pit formation in open tracheal system	biological process	0.02158497593	
GO:1990511	piRNA biosynthetic process	biological process	0.02158497593	
GO:2000274	regulation of epithelial cell migration, open tracheal system	biological process	0.02158497593	
GO:0060560	developmental growth involved in morphogenesis	biological process	0.02229988276	
GO:0004714	transmembrane receptor protein tyrosine kinase activity	molecular function	0.02243102676	
GO:0042995	cell projection	cellular component	0.02276848392	
GO:0050790	regulation of catalytic activity	biological process	0.02278169689	
GO:0022416	chaeta development	biological process	0.02295202045	
GO:0030426	growth cone	cellular component	0.02323683814	
GO:0000075	cell cycle checkpoint	biological process	0.02471928595	
GO:0009950	dorsal/ventral axis specification	biological process	0.02471928595	
GO:0030863	cortical cytoskeleton	cellular component	0.02472628717	
GO:0002052	positive regulation of neuroblast proliferation	biological process	0.02499602426	
GO:0042059	negative regulation of epidermal growth factor receptor signaling pathway	biological process	0.02499602426	
GO:0043954	cellular component maintenance	biological process	0.02499602426	
GO:0045860	positive regulation of protein kinase activity	biological process	0.02499602426	
GO:0070828	heterochromatin organization	biological process	0.02499602426	

GO:1901185	negative regulation of ERBB signaling pathway	biological process	0.02499602426	
GO:0019207	kinase regulator activity	molecular function	0.02533036803	
GO:0000902	cell morphogenesis	biological process	0.02633203508	
GO:0000076	DNA replication checkpoint	biological process	0.02656247498	
GO:0007362	terminal region determination	biological process	0.02656247498	
GO:0031570	DNA integrity checkpoint	biological process	0.02656247498	
GO:0045167	asymmetric protein localization involved in cell fate determination	biological process	0.02656247498	
GO:0046426	negative regulation of receptor signaling pathway via JAK-STAT	biological process	0.02656247498	
GO:0048639	positive regulation of developmental growth	biological process	0.02656247498	
GO:0051310	metaphase plate congression	biological process	0.02656247498	
GO:1904893	negative regulation of receptor signaling pathway via STAT	biological process	0.02656247498	
GO:0061982	meiosis I cell cycle process	biological process	0.02695024767	
GO:0035102	PRC1 complex	cellular component	0.02765507168	
GO:0120025	plasma membrane bounded cell projection	cellular component	0.02784817406	
GO:0000307	cyclin-dependent protein kinase holoenzyme complex	cellular component	0.02890865189	
GO:0016772	transferase activity, transferring phosphorus-containing groups	molecular function		0.02916476497
GO:0019199	transmembrane receptor protein kinase activity	molecular function		0.02916476497
GO:0036094	small molecule binding	molecular function		0.02916476497
GO:0016324	apical plasma membrane	cellular component	0.02971493677	
GO:0016327	apicolateral plasma membrane	cellular component	0.02971493677	
GO:1901991	negative regulation of mitotic cell cycle phase transition	biological process	0.02972577126	
GO:0030718	germ-line stem cell population maintenance	biological process	0.02989360888	
GO:0016055	Wnt signaling pathway	biological process	0.03050250343	
GO:0045785	positive regulation of cell adhesion	biological process	0.03050250343	
GO:1905114	cell surface receptor signaling pathway involved in cell-cell signaling	biological process	0.03050250343	
GO:0019887	protein kinase regulator activity	molecular function	0.03053294424	
GO:0030723	ovarian fusome organization	biological process	0.0305603422	
GO:0035203	regulation of lamellocyte differentiation	biological process	0.0305603422	
GO:0035215	genital disc development	biological process	0.0305603422	
GO:0035330	regulation of hippo signaling	biological process	0.0305603422	
GO:0042766	nucleosome mobilization	biological process	0.0305603422	
GO:0044772	mitotic cell cycle phase transition	biological process	0.0305603422	
GO:0046427	positive regulation of receptor signaling pathway via JAK-STAT	biological process	0.0305603422	

GO:0048841	regulation of axon extension involved in axon guidance	biological process	0.0305603422	
GO:0060232	delamination	biological process	0.0305603422	
GO:1904894	positive regulation of receptor signaling pathway via STAT	biological process	0.0305603422	
GO:2000737	negative regulation of stem cell differentiation	biological process	0.0305603422	
GO:0001221	transcription cofactor binding	molecular function	0.03057745278	
GO:0030427	site of polarized growth	cellular component	0.03189715307	
GO:0045927	positive regulation of growth	biological process	0.0331102548	
GO:1901988	negative regulation of cell cycle phase transition	biological process	0.03326929706	
GO:0004674	protein serine/threonine kinase activity	molecular function		0.03341812203
GO:0034333	adherens junction assembly	biological process	0.03386355407	
GO:0048100	wing disc anterior/posterior pattern formation	biological process	0.03386355407	
GO:0070593	dendrite self-avoidance	biological process	0.03386355407	
GO:0007043	cell-cell junction assembly	biological process	0.03405205062	
GO:0031208	POZ domain binding	molecular function	0.03418961364	
GO:0006305	DNA alkylation	biological process	0.03538015606	
GO:0006306	DNA methylation	biological process	0.03538015606	
GO:0007157	heterophilic cell-cell adhesion via plasma membrane cell adhesion molecul	biological process	0.03538015606	
GO:0007355	anterior region determination	biological process	0.03538015606	
GO:0007376	cephalic furrow formation	biological process	0.03538015606	
GO:0007381	specification of segmental identity, labial segment	biological process	0.03538015606	
GO:0007382	specification of segmental identity, maxillary segment	biological process	0.03538015606	
GO:0007383	specification of segmental identity, antennal segment	biological process	0.03538015606	
GO:0007421	stomatogastric nervous system development	biological process	0.03538015606	
GO:0031099	regeneration	biological process	0.03538015606	
GO:0031345	negative regulation of cell projection organization	biological process	0.03538015606	
GO:0034114	regulation of heterotypic cell-cell adhesion	biological process	0.03538015606	
GO:0034116	positive regulation of heterotypic cell-cell adhesion	biological process	0.03538015606	
GO:0035038	female pronucleus assembly	biological process	0.03538015606	
GO:0035155	negative regulation of terminal cell fate specification, open tracheal system	biological process	0.03538015606	
GO:0035157	negative regulation of fusion cell fate specification	biological process	0.03538015606	
GO:0035260	internal genitalia morphogenesis	biological process	0.03538015606	
GO:0038007	netrin-activated signaling pathway	biological process	0.03538015606	
GO:0042062	long-term strengthening of neuromuscular junction	biological process	0.03538015606	

GO:0042693	muscle cell fate commitment	biological process	0.03538015606	
GO:0045685	regulation of glial cell differentiation	biological process	0.03538015606	
GO:0045687	positive regulation of glial cell differentiation	biological process	0.03538015606	
GO:0048511	rhythmic process	biological process	0.03538015606	
GO:0048936	peripheral nervous system neuron axonogenesis	biological process	0.03538015606	
GO:0060233	oenocyte delamination	biological process	0.03538015606	
GO:0071921	cohesin loading	biological process	0.03538015606	
GO:1901989	positive regulation of cell cycle phase transition	biological process	0.03538015606	
GO:1901992	positive regulation of mitotic cell cycle phase transition	biological process	0.03538015606	
GO:1902337	regulation of apoptotic process involved in morphogenesis	biological process	0.03538015606	
GO:1902339	positive regulation of apoptotic process involved in morphogenesis	biological process	0.03538015606	
GO:0007313	maternal specification of dorsal/ventral axis, oocyte, soma encoded	biological process	0.03584587043	
GO:0016202	regulation of striated muscle tissue development	biological process	0.03584587043	
GO:0038004	epidermal growth factor receptor ligand maturation	biological process	0.03584587043	
GO:0043703	photoreceptor cell fate determination	biological process	0.03584587043	
GO:0046665	amnioserosa maintenance	biological process	0.03584587043	
GO:0048645	animal organ formation	biological process	0.03584587043	
GO:0048854	brain morphogenesis	biological process	0.03584587043	
GO:0060914	heart formation	biological process	0.03584587043	
GO:1901861	regulation of muscle tissue development	biological process	0.03584587043	
GO:1902292	cell cycle DNA replication initiation	biological process	0.03584587043	
GO:1902315	nuclear cell cycle DNA replication initiation	biological process	0.03584587043	
GO:1902975	mitotic DNA replication initiation	biological process	0.03584587043	
GO:1904666	regulation of ubiquitin protein ligase activity	biological process	0.03584587043	
GO:0006323	DNA packaging	biological process	0.03612055155	
GO:0090287	regulation of cellular response to growth factor stimulus	biological process	0.03612055155	
GO:0043900	regulation of multi-organism process	biological process	0.03612604315	
GO:0008344	adult locomotory behavior	biological process	0.03695366106	
GO:0016569	covalent chromatin modification	biological process	0.03722897229	
GO:0016570	histone modification	biological process	0.03722897229	
GO:0031010	ISWI-type complex	cellular component	0.03743806775	
GO:0006364	rRNA processing	biological process	0.03831645941	
GO:0044087	regulation of cellular component biogenesis	biological process	0.03888470424	

GO:0008285	negative regulation of cell population proliferation	biological process	0.03888691072	
GO:0030686	90S preribosome	cellular component	0.03906162009	
GO:0003007	heart morphogenesis	biological process	0.03997015597	
GO:0008594	photoreceptor cell morphogenesis	biological process	0.03997015597	
GO:0016572	histone phosphorylation	biological process	0.03997015597	
GO:0035285	appendage segmentation	biological process	0.03997015597	
GO:0036011	imaginal disc-derived leg segmentation	biological process	0.03997015597	
GO:0060438	trachea development	biological process	0.03997015597	
GO:0007390	germ-band shortening	biological process	0.0401871888	
GO:0035212	cell competition in a multicellular organism	biological process	0.0401871888	
GO:0051101	regulation of DNA binding	biological process	0.0401871888	
GO:0007472	wing disc morphogenesis	biological process	0.04119322802	
GO:0003008	system process	biological process	0.04150098814	
GO:0001736	establishment of planar polarity	biological process	0.04247629732	
GO:0007164	establishment of tissue polarity	biological process	0.04247629732	
GO:0030097	hemopoiesis	biological process	0.04247629732	
GO:0044770	cell cycle phase transition	biological process	0.04247629732	
GO:0010562	positive regulation of phosphorus metabolic process	biological process	0.0433255395	
GO:0045937	positive regulation of phosphate metabolic process	biological process	0.0433255395	
GO:0045132	meiotic chromosome segregation	biological process	0.04342460415	
GO:0051124	synaptic growth at neuromuscular junction	biological process	0.04342460415	
GO:0090288	negative regulation of cellular response to growth factor stimulus	biological process	0.04342460415	
GO:0098631	cell adhesion mediator activity	molecular function	0.04375386551	
GO:0000118	histone deacetylase complex	cellular component	0.0453417026	
GO:0045995	regulation of embryonic development	biological process	0.0474906856	
GO:0035097	histone methyltransferase complex	cellular component	0.04778683196	
GO:0016538	cyclin-dependent protein serine/threonine kinase regulator activity	molecular function	0.04902529885	
GO:0009986	cell surface	cellular component	0.04908171234	
GO:0007369	gastrulation	biological process	0.04982781844	
GO:0042067	establishment of ommatidial planar polarity	biological process	0.04982781844	

GO	name	GO type	p-value FDR *D. melanogaster*	p-value FDR *D. virilis*
GO:0003735	structural constituent of ribosome	molecular function	4.71E-73	3.12E-07
GO:0043043	peptide biosynthetic process	biological process	6.21E-71	1.12E-06
GO:0006518	peptide metabolic process	biological process	1.28E-68	1.12E-06
GO:0006412	translation	biological process	1.28E-68	1.96E-06
GO:0043604	amide biosynthetic process	biological process	1.62E-67	5.00E-06
GO:0043603	cellular amide metabolic process	biological process	1.59E-61	9.12E-06
GO:0005198	structural molecule activity	molecular function	9.43E-53	8.84E-09
GO:1901566	organonitrogen compound biosynthetic process	biological process	2.70E-53	1.05E-06
GO:0009058	biosynthetic process	biological process	9.01E-39	1.88E-05
GO:1901576	organic substance biosynthetic process	biological process	1.98E-36	4.92E-05
GO:0044249	cellular biosynthetic process	biological process	1.92E-36	5.11E-05
GO:0044271	cellular nitrogen compound biosynthetic process	biological process	1.94E-37	5.62E-04
GO:0005739	mitochondrion	cellular component	1.88E-38	4.88E-02
GO:0034645	cellular macromolecule biosynthetic process	biological process	1.73E-35	2.25E-04
GO:0005840	ribosome	cellular component	3.15E-32	8.09E-06
GO:0008152	metabolic process	biological process	1.59E-20	1.25E-09
GO:0009059	macromolecule biosynthetic process	biological process	8.22E-27	2.99E-02
GO:0044281	small molecule metabolic process	biological process	6.89E-21	1.12E-06
GO:0019752	carboxylic acid metabolic process	biological process	4.30E-22	5.58E-05
GO:0043436	oxoacid metabolic process	biological process	3.46E-21	2.51E-05
GO:0006082	organic acid metabolic process	biological process	8.19E-21	2.51E-05
GO:1901564	organonitrogen compound metabolic process	biological process	8.19E-21	1.29E-04
GO:0003824	catalytic activity	molecular function	5.60E-17	3.12E-07
GO:0034641	cellular nitrogen compound metabolic process	biological process	1.52E-19	3.59E-02
GO:0016491	oxidoreductase activity	molecular function	3.46E-14	2.81E-07
GO:0071704	organic substance metabolic process	biological process	8.47E-17	2.52E-04
GO:0006520	cellular amino acid metabolic process	biological process	9.84E-13	1.61E-03
GO:0015078	proton transmembrane transporter activity	molecular function	8.74E-09	9.39E-07
GO:0006807	nitrogen compound metabolic process	biological process	3.04E-12	0.003180212508
GO:1902600	proton transmembrane transport	biological process	5.43E-09	4.55E-06
GO:0008061	chitin binding	molecular function	9.52E-11	0.001780169847
GO:0044238	primary metabolic process	biological process	2.44E-11	4.59E-02

GO:0044237	cellular metabolic process	biological process	7.20E-10	0.01518481445
GO:0033178	proton-transporting two-sector ATPase complex, catalytic domain	cellular component	6.48E-06	8.18E-06
GO:0055114	oxidation-reduction process	biological process	2.42E-05	1.90E-05
GO:0055085	transmembrane transport	biological process	1.24E-04	1.90E-05
GO:0015077	monovalent inorganic cation transmembrane transporter activity	molecular function	2.64E-04	5.94E-05
GO:0098655	cation transmembrane transport	biological process	0.003685908629	2.22E-05
GO:0098660	inorganic ion transmembrane transport	biological process	0.003801638484	2.51E-05
GO:0098662	inorganic cation transmembrane transport	biological process	0.004768138126	2.22E-05
GO:0009678	pyrophosphate hydrolysis-driven proton transmembrane transporter activity	molecular function	9.62E-06	0.01119516344
GO:0044769	ATPase activity, coupled to transmembrane movement of ions, rotational m	molecular function	9.62E-06	1.12E-02
GO:0046961	proton-transporting ATPase activity, rotational mechanism	molecular function	9.62E-06	1.12E-02
GO:0009145	purine nucleoside triphosphate biosynthetic process	biological process	1.82E-04	7.22E-04
GO:0009205	purine ribonucleoside triphosphate metabolic process	biological process	1.82E-04	7.22E-04
GO:0009206	purine ribonucleoside triphosphate biosynthetic process	biological process	1.82E-04	7.22E-04
GO:0015672	monovalent inorganic cation transport	biological process	5.54E-05	2.64E-03
GO:0009144	purine nucleoside triphosphate metabolic process	biological process	2.77E-04	7.22E-04
GO:0098796	membrane protein complex	cellular component	1.83E-03	1.50E-04
GO:0022804	active transmembrane transporter activity	molecular function	1.85E-03	2.88E-04
GO:0009199	ribonucleoside triphosphate metabolic process	biological process	6.06E-04	1.06E-03
GO:0009201	ribonucleoside triphosphate biosynthetic process	biological process	6.06E-04	1.06E-03
GO:0015985	energy coupled proton transport, down electrochemical gradient	biological process	1.05E-04	8.68E-03
GO:0015986	ATP synthesis coupled proton transport	biological process	1.05E-04	8.68E-03
GO:0046933	proton-transporting ATP synthase activity, rotational mechanism	molecular function	1.23E-04	7.40E-03
GO:0006754	ATP biosynthetic process	biological process	1.71E-04	8.68E-03
GO:1901135	carbohydrate derivative metabolic process	biological process	6.97E-04	2.41E-03
GO:0009142	nucleoside triphosphate biosynthetic process	biological process	1.74E-03	1.06E-03
GO:0009141	nucleoside triphosphate metabolic process	biological process	1.04E-03	2.13E-03
GO:0009260	ribonucleotide biosynthetic process	biological process	1.15E-04	2.86E-02
GO:0005215	transporter activity	molecular function	2.25E-03	1.78E-03
GO:0015252	proton channel activity	molecular function	5.74E-04	7.40E-03
GO:0046390	ribose phosphate biosynthetic process	biological process	1.60E-04	2.86E-02
GO:0034220	ion transmembrane transport	biological process	3.83E-02	1.40E-04
GO:0046034	ATP metabolic process	biological process	1.57E-04	3.75E-02

GO:0042626	ATPase-coupled transmembrane transporter activity	molecular function	8.14E-04	0.007403206122
GO:0015399	primary active transmembrane transporter activity	molecular function	0.001998922142	0.003343515607
GO:0042302	structural constituent of cuticle	molecular function	0.0001661145402	4.25E-02
GO:0022857	transmembrane transporter activity	molecular function	0.01433850954	0.001218864239
GO:0033180	proton-transporting V-type ATPase, V1 domain	cellular component	0.0019177334	0.02227722038
GO:0006811	ion transport	biological process	0.005904991124	0.01019448124
GO:0022853	active ion transmembrane transporter activity	molecular function	0.006306461199	0.01464037518
GO:0045261	proton-transporting ATP synthase complex, catalytic core F(1)	cellular component	0.01057661459	0.01502690943
GO:0005576	extracellular region	cellular component	0.005074127193	0.04878956165
GO:0090407	organophosphate biosynthetic process	biological process	0.02386841205	0.01080294792
GO:0006812	cation transport	biological process	0.04087218599	0.00904012799
GO:0016769	transferase activity, transferring nitrogenous groups	molecular function	0.0496509783	0.008346280963
GO:0044391	ribosomal subunit	cellular component	6.22E-73	
GO:0098798	mitochondrial protein complex	cellular component	3.40E-49	
GO:0015934	large ribosomal subunit	cellular component	9.22E-49	
GO:0032543	mitochondrial translation	biological process	2.10E-42	
GO:0000315	organellar large ribosomal subunit	cellular component	2.43E-32	
GO:0005762	mitochondrial large ribosomal subunit	cellular component	2.43E-32	
GO:0022626	cytosolic ribosome	cellular component	4.57E-32	
GO:0002181	cytoplasmic translation	biological process	3.50E-30	
GO:0015935	small ribosomal subunit	cellular component	1.01E-23	
GO:1990904	ribonucleoprotein complex	cellular component	2.31E-23	
GO:0022625	cytosolic large ribosomal subunit	cellular component	2.38E-18	
GO:0000314	organellar small ribosomal subunit	cellular component	1.43E-13	
GO:0005763	mitochondrial small ribosomal subunit	cellular component	1.43E-13	
GO:0044282	small molecule catabolic process	biological process	2.19E-12	
GO:0016054	organic acid catabolic process	biological process	3.78E-12	
GO:0046395	carboxylic acid catabolic process	biological process	3.78E-12	
GO:0022627	cytosolic small ribosomal subunit	cellular component	6.40E-11	
GO:0098800	inner mitochondrial membrane protein complex	cellular component	1.52E-10	
GO:0019843	rRNA binding	molecular function	4.29E-10	
GO:0009063	cellular amino acid catabolic process	biological process	6.17E-10	
GO:0016469	proton-transporting two-sector ATPase complex	cellular component	7.70E-10	

GO:0032787	monocarboxylic acid metabolic process	biological process	1.81E-09	
GO:1901606	alpha-amino acid catabolic process	biological process	1.55E-08	
GO:1901605	alpha-amino acid metabolic process	biological process	2.13E-08	
GO:0034470	ncRNA processing	biological process	3.11E-08	
GO:0044283	small molecule biosynthetic process	biological process	3.54E-08	
GO:0034660	ncRNA metabolic process	biological process	7.28E-08	
GO:0033176	proton-transporting V-type ATPase complex	cellular component	8.14E-08	
GO:0006749	glutathione metabolic process	biological process	9.02E-08	
GO:1990204	oxidoreductase complex	cellular component	1.13E-07	
GO:0098803	respiratory chain complex	cellular component	1.23E-07	
GO:0009081	branched-chain amino acid metabolic process	biological process	1.30E-07	
GO:0033181	plasma membrane proton-transporting V-type ATPase complex	cellular component	1.90E-07	
GO:0006631	fatty acid metabolic process	biological process	2.25E-07	
GO:0016072	rRNA metabolic process	biological process	2.58E-07	
GO:0006364	rRNA processing	biological process	4.77E-07	
GO:0006575	cellular modified amino acid metabolic process	biological process	5.30E-07	
GO:0005747	mitochondrial respiratory chain complex I	cellular component	5.44E-07	
GO:0030964	NADH dehydrogenase complex	cellular component	5.44E-07	
GO:0045271	respiratory chain complex I	cellular component	5.44E-07	
GO:0006790	sulfur compound metabolic process	biological process	8.60E-07	
GO:0009083	branched-chain amino acid catabolic process	biological process	9.35E-07	
GO:0006399	tRNA metabolic process	biological process	2.13E-06	
GO:0000027	ribosomal large subunit assembly	biological process	2.94E-06	
GO:0019538	protein metabolic process	biological process	8.68E-06	
GO:0016616	oxidoreductase activity, acting on the CH-OH group of donors, NAD or NA	molecular function	9.62E-06	
GO:0022613	ribonucleoprotein complex biogenesis	biological process	1.98E-05	
GO:0016874	ligase activity	molecular function	2.26E-05	
GO:0062129	chitin-based extracellular matrix	cellular component	2.34E-05	
GO:0016765	transferase activity, transferring alkyl or aryl (other than methyl) groups	molecular function	2.36E-05	
GO:0009150	purine ribonucleotide metabolic process	biological process	3.06E-05	
GO:0033108	mitochondrial respiratory chain complex assembly	biological process	3.48E-05	
GO:0004180	carboxypeptidase activity	molecular function	3.99E-05	
GO:0009259	ribonucleotide metabolic process	biological process	5.73E-05	

GO:0031012	extracellular matrix	cellular component	6.74E-05	
GO:0072521	purine-containing compound metabolic process	biological process	6.77E-05	
GO:0019842	vitamin binding	molecular function		7.03E-05
GO:0009152	purine ribonucleotide biosynthetic process	biological process	7.13E-05	
GO:0019693	ribose phosphate metabolic process	biological process	8.57E-05	
GO:0006163	purine nucleotide metabolic process	biological process	8.58E-05	
GO:0006091	generation of precursor metabolites and energy	biological process	8.99E-05	
GO:0016614	oxidoreductase activity, acting on CH-OH group of donors	molecular function	9.85E-05	
GO:0019395	fatty acid oxidation	biological process	0.0001046221833	
GO:0044272	sulfur compound biosynthetic process	biological process	0.0001171261428	
GO:0042273	ribosomal large subunit biogenesis	biological process	0.0001386986191	
GO:0034440	lipid oxidation	biological process	0.0001452860779	
GO:0006164	purine nucleotide biosynthetic process	biological process	0.0001494080021	
GO:0044085	cellular component biogenesis	biological process	0.0001856725122	
GO:0055086	nucleobase-containing small molecule metabolic process	biological process	0.000199043776	
GO:0019829	ATPase-coupled cation transmembrane transporter activity	molecular function	0.0002180134635	
GO:0005214	structural constituent of chitin-based cuticle	molecular function	0.0002253155765	
GO:0017171	serine hydrolase activity	molecular function	0.0002556510454	
GO:0072329	monocarboxylic acid catabolic process	biological process	0.0002564189099	
GO:0043228	non-membrane-bounded organelle	cellular component		0.000261781106
GO:0043232	intracellular non-membrane-bounded organelle	cellular component		0.000261781106
GO:0006753	nucleoside phosphate metabolic process	biological process	0.0002619868391	
GO:0000221	vacuolar proton-transporting V-type ATPase, V1 domain	cellular component	0.0002785858967	
GO:0010257	NADH dehydrogenase complex assembly	biological process	0.0003298477803	
GO:0032981	mitochondrial respiratory chain complex I assembly	biological process	0.0003298477803	
GO:0008033	tRNA processing	biological process	0.0003825697999	
GO:0072522	purine-containing compound biosynthetic process	biological process	0.000387143632	
GO:0006551	leucine metabolic process	biological process	0.0004007967166	
GO:0006573	valine metabolic process	biological process	0.0004007967166	
GO:0070071	proton-transporting two-sector ATPase complex assembly	biological process	0.0004013200695	
GO:0016471	vacuolar proton-transporting V-type ATPase complex	cellular component	0.0004091865977	
GO:0008150	biological_process	biological process		0.0005059512276
GO:0008233	peptidase activity	molecular function	0.0005431395459	

GO:0004364	glutathione transferase activity	molecular function	0.0005743363221	
GO:0042625	ATPase-coupled ion transmembrane transporter activity	molecular function	0.0005743363221	
GO:0009117	nucleotide metabolic process	biological process	0.0005815834619	
GO:0033177	proton-transporting two-sector ATPase complex, proton-transporting domai	cellular component	0.0005890047867	
GO:0016861	intramolecular oxidoreductase activity, interconverting aldoses and ketoses	molecular function	0.000598709471	
GO:0009062	fatty acid catabolic process	biological process	0.0006971776712	
GO:0006635	fatty acid beta-oxidation	biological process	0.0007406368421	
GO:0008236	serine-type peptidase activity	molecular function	0.0009053898173	
GO:0022890	inorganic cation transmembrane transporter activity	molecular function		0.001111657327
GO:0006030	chitin metabolic process	biological process		0.001141836046
GO:0006022	aminoglycan metabolic process	biological process		0.001247902434
GO:0016810	hydrolase activity, acting on carbon-nitrogen (but not peptide) bonds	molecular function	0.001338585225	
GO:0006040	amino sugar metabolic process	biological process		0.001428513299
GO:1901071	glucosamine-containing compound metabolic process	biological process		0.001428513299
GO:0016053	organic acid biosynthetic process	biological process	0.001432578695	
GO:0046394	carboxylic acid biosynthetic process	biological process	0.001432578695	
GO:0006839	mitochondrial transport	biological process	0.001649529561	
GO:0008970	phospholipase A1 activity	molecular function	0.001913812403	
GO:0016811	hydrolase activity, acting on carbon-nitrogen (but not peptide) bonds, in lin	molecular function	0.002019041973	
GO:0005777	peroxisome	cellular component	0.002154455465	
GO:0008010	structural constituent of chitin-based larval cuticle	molecular function	0.002252399852	
GO:0008324	cation transmembrane transporter activity	molecular function		0.002476469495
GO:0040003	chitin-based cuticle development	biological process	0.002541176052	
GO:0042579	microbody	cellular component	0.00281720241	
GO:0006552	leucine catabolic process	biological process	0.00295625856	
GO:0006574	valine catabolic process	biological process	0.00295625856	
GO:0009987	cellular process	biological process		0.002957370882
GO:0004252	serine-type endopeptidase activity	molecular function	0.003346737478	
GO:0042335	cuticle development	biological process	0.003467218044	
GO:0000062	fatty-acyl-CoA binding	molecular function	0.003781994656	
GO:1901567	fatty acid derivative binding	molecular function	0.003781994656	
GO:0045851	pH reduction	biological process	0.003864591794	
GO:0051452	intracellular pH reduction	biological process	0.003864591794	

GO:0009165	nucleotide biosynthetic process	biological process	0.003912290758	
GO:1901293	nucleoside phosphate biosynthetic process	biological process	0.004128769852	
GO:0030170	pyridoxal phosphate binding	molecular function		0.004232591179
GO:0070279	vitamin B6 binding	molecular function		0.004232591179
GO:0004175	endopeptidase activity	molecular function	0.004432912034	
GO:0004181	metallocarboxypeptidase activity	molecular function	0.004485445737	
GO:0006400	tRNA modification	biological process	0.004909433931	
GO:0009068	aspartate family amino acid catabolic process	biological process	0.005162601373	
GO:0140101	catalytic activity, acting on a tRNA	molecular function	0.005452433944	
GO:0000462	maturation of SSU-rRNA from tricistronic rRNA transcript (SSU-rRNA, 5.	biological process	0.005454184733	
GO:0008097	5S rRNA binding	molecular function	0.005463126611	
GO:0016787	hydrolase activity	molecular function	0.00570035844	
GO:0006743	ubiquinone metabolic process	biological process	0.005871119209	
GO:0006744	ubiquinone biosynthetic process	biological process	0.005871119209	
GO:1901661	quinone metabolic process	biological process	0.005871119209	
GO:1901663	quinone biosynthetic process	biological process	0.005871119209	
GO:0044267	cellular protein metabolic process	biological process	0.006036567387	
GO:0008238	exopeptidase activity	molecular function	0.006306461199	
GO:0120227	acyl-CoA binding	molecular function	0.006306461199	
GO:1901137	carbohydrate derivative biosynthetic process	biological process	0.006477464704	
GO:0070013	intracellular organelle lumen	cellular component	0.007006197721	
GO:0008237	metallopeptidase activity	molecular function	0.007040024529	
GO:0051453	regulation of intracellular pH	biological process	0.007597684389	
GO:0070585	protein localization to mitochondrion	biological process	0.007755423853	
GO:0072655	establishment of protein localization to mitochondrion	biological process	0.007755423853	
GO:0031974	membrane-enclosed lumen	cellular component	0.007847114258	
GO:0043233	organelle lumen	cellular component	0.007847114258	
GO:0043170	macromolecule metabolic process	biological process	0.008065305058	
GO:0033293	monocarboxylic acid binding	molecular function	0.008294015515	
GO:0015318	inorganic molecular entity transmembrane transporter activity	molecular function		0.008307471494
GO:0005783	endoplasmic reticulum	cellular component		0.008339105447
GO:0008483	transaminase activity	molecular function		0.008346280963
GO:0045039	protein insertion into mitochondrial inner membrane	biological process	0.008413266061	

GO:0006885	regulation of pH	biological process	0.008507783905	
GO:0030641	regulation of cellular pH	biological process	0.008507783905	
GO:0044255	cellular lipid metabolic process	biological process	0.008507783905	
GO:0008235	metalloexopeptidase activity	molecular function	0.009118843356	
GO:0051204	protein insertion into mitochondrial membrane	biological process	0.009284119557	
GO:0006414	translational elongation	biological process	0.01048456506	
GO:0000313	organellar ribosome	cellular component	0.01057661459	
GO:0005761	mitochondrial ribosome	cellular component	0.01057661459	
GO:0006591	ornithine metabolic process	biological process	0.01087918789	
GO:0006750	glutathione biosynthetic process	biological process	0.01087918789	
GO:0019184	nonribosomal peptide biosynthetic process	biological process	0.01087918789	
GO:0070070	proton-transporting V-type ATPase complex assembly	biological process	0.01091329543	
GO:0070072	vacuolar proton-transporting V-type ATPase complex assembly	biological process	0.01091329543	
GO:0008535	respiratory chain complex IV assembly	biological process	0.01238468104	
GO:0042398	cellular modified amino acid biosynthetic process	biological process	0.01238468104	
GO:0005736	RNA polymerase I complex	cellular component	0.0137727307	
GO:0035384	thioester biosynthetic process	biological process	0.01403994531	
GO:0071616	acyl-CoA biosynthetic process	biological process	0.01403994531	
GO:0004656	procollagen-proline 4-dioxygenase activity	molecular function		0.01464037518
GO:0009055	electron transfer activity	molecular function		0.01464037518
GO:0015075	ion transmembrane transporter activity	molecular function		0.01464037518
GO:0019798	procollagen-proline dioxygenase activity	molecular function		0.01464037518
GO:0031406	carboxylic acid binding	molecular function		0.01464037518
GO:0031543	peptidyl-proline dioxygenase activity	molecular function		0.01464037518
GO:0031545	peptidyl-proline 4-dioxygenase activity	molecular function		0.01464037518
GO:0043177	organic acid binding	molecular function		0.01464037518
GO:0005759	mitochondrial matrix	cellular component	0.01618402047	
GO:0030684	preribosome	cellular component	0.01633881831	
GO:0044242	cellular lipid catabolic process	biological process	0.01642262549	
GO:0030490	maturation of SSU-rRNA	biological process	0.01810870988	
GO:0005587	collagen type IV trimer	cellular component	0.01853272113	
GO:0098642	network-forming collagen trimer	cellular component	0.01853272113	
GO:0098651	basement membrane collagen trimer	cellular component	0.01853272113	

GO:0000177	cytoplasmic exosome (RNase complex)	cellular component	0.01864468139	
GO:0000220	vacuolar proton-transporting V-type ATPase, V0 domain	cellular component	0.01864468139	
GO:0003012	muscle system process	biological process	0.01875134577	
GO:0004185	serine-type carboxypeptidase activity	molecular function	0.01912242894	
GO:0016624	oxidoreductase activity, acting on the aldehyde or oxo group of donors, disu	molecular function	0.01912242894	
GO:0036042	long-chain fatty acyl-CoA binding	molecular function	0.01912242894	
GO:0070181	small ribosomal subunit rRNA binding	molecular function	0.01912242894	
GO:0016042	lipid catabolic process	biological process	0.01930424834	
GO:0016407	acetyltransferase activity	molecular function		0.0193687732
GO:0000178	exosome (RNase complex)	cellular component	0.01983238558	
GO:0071722	detoxification of arsenic-containing substance	biological process	0.01989735232	
GO:0006094	gluconeogenesis	biological process	0.0203378684	
GO:0019319	hexose biosynthetic process	biological process	0.0203378684	
GO:0008652	cellular amino acid biosynthetic process	biological process	0.0205590804	
GO:0006629	lipid metabolic process	biological process	0.02171804837	
GO:0007035	vacuolar acidification	biological process	0.02217951741	
GO:0045240	dihydrolipoyl dehydrogenase complex	cellular component	0.02279992911	
GO:0003723	RNA binding	molecular function	0.02296568616	
GO:0009066	aspartate family amino acid metabolic process	biological process	0.02324629639	
GO:0005506	iron ion binding	molecular function		0.02343903549
GO:0033866	nucleoside bisphosphate biosynthetic process	biological process	0.02352238892	
GO:0034030	ribonucleoside bisphosphate biosynthetic process	biological process	0.02352238892	
GO:0034033	purine nucleoside bisphosphate biosynthetic process	biological process	0.02352238892	
GO:0009451	RNA modification	biological process	0.02365637099	
GO:0000470	maturation of LSU-rRNA	biological process	0.02385845099	
GO:0017004	cytochrome complex assembly	biological process	0.02385845099	
GO:0005730	nucleolus	cellular component	0.02467463525	
GO:0033179	proton-transporting V-type ATPase, V0 domain	cellular component	0.02480265	
GO:0002814	negative regulation of biosynthetic process of antibacterial peptides active a	biological process	0.0255810848	
GO:0022900	electron transport chain	biological process	0.0262425427	
GO:0019205	nucleobase-containing compound kinase activity	molecular function		0.02648357792
GO:1901607	alpha-amino acid biosynthetic process	biological process	0.02666350585	
GO:0016705	oxidoreductase activity, acting on paired donors, with incorporation or redu	molecular function		0.0280205798

GO:0016747	transferase activity, transferring acyl groups other than amino-acyl groups	molecular function		0.0280205798
GO:0045239	tricarboxylic acid cycle enzyme complex	cellular component	0.02827301997	
GO:1905354	exoribonuclease complex	cellular component	0.02827301997	
GO:0000176	nuclear exosome (RNase complex)	cellular component	0.02843422115	
GO:0005753	mitochondrial proton-transporting ATP synthase complex	cellular component	0.02843422115	
GO:0045259	proton-transporting ATP synthase complex	cellular component	0.02843422115	
GO:0008080	N-acetyltransferase activity	molecular function		0.02852329209
GO:0007006	mitochondrial membrane organization	biological process	0.02938717754	
GO:0007007	inner mitochondrial membrane organization	biological process	0.03037009605	
GO:0004576	oligosaccharyl transferase activity	molecular function		0.03105609791
GO:0016776	phosphotransferase activity, phosphate group as acceptor	molecular function		0.03105609791
GO:0016627	oxidoreductase activity, acting on the CH-CH group of donors	molecular function	0.03203123429	
GO:0016706	2-oxoglutarate-dependent dioxygenase activity	molecular function		0.03225799337
GO:1990542	mitochondrial transmembrane transport	biological process	0.03256368185	
GO:0004090	carbonyl reductase (NADPH) activity	molecular function	0.03522793176	
GO:0005504	fatty acid binding	molecular function	0.035555088	
GO:0006936	muscle contraction	biological process	0.03726772367	
GO:0006081	cellular aldehyde metabolic process	biological process	0.03760516377	
GO:0016410	N-acyltransferase activity	molecular function		0.03778679892
GO:0072593	reactive oxygen species metabolic process	biological process	0.03866436421	
GO:0004035	alkaline phosphatase activity	molecular function	0.0386851109	
GO:0006633	fatty acid biosynthetic process	biological process	0.03919230145	
GO:0016746	transferase activity, transferring acyl groups	molecular function		0.04248438207
GO:0070069	cytochrome complex	cellular component	0.04316291569	
GO:0000276	mitochondrial proton-transporting ATP synthase complex, coupling factor F	cellular component	0.04353596999	
GO:0045263	proton-transporting ATP synthase complex, coupling factor F(o)	cellular component	0.04353596999	
GO:0009069	serine family amino acid metabolic process	biological process	0.04531735226	
GO:0004046	aminoacylase activity	molecular function	0.04595440937	
GO:0070008	serine-type exopeptidase activity	molecular function	0.04595440937	
GO:0055067	monovalent inorganic cation homeostasis	biological process	0.04632853321	
GO:0016903	oxidoreductase activity, acting on the aldehyde or oxo group of donors	molecular function	0.04696247282	
GO:0002787	negative regulation of antibacterial peptide production	biological process	0.04762779815	
GO:0002806	negative regulation of antimicrobial peptide biosynthetic process	biological process	0.04762779815	

GO:0002809	negative regulation of antibacterial peptide biosynthetic process	biological process	0.04762779815	
GO:0034475	U4 snRNA 3'-end processing	biological process	0.04762779815	
GO:0000275	mitochondrial proton-transporting ATP synthase complex, catalytic sector F	cellular component	0.04800918052	
GO:0005581	collagen trimer	cellular component	0.04800918052	
GO:0005756	mitochondrial proton-transporting ATP synthase, central stalk	cellular component	0.04800918052	
GO:0005903	brush border	cellular component	0.04800918052	
GO:0031314	extrinsic component of mitochondrial inner membrane	cellular component	0.04800918052	
GO:0045269	proton-transporting ATP synthase, central stalk	cellular component	0.04800918052	
GO:0098862	cluster of actin-based cell projections	cellular component	0.04800918052	
GO:0005622	intracellular	cellular component		0.04878956165
GO:1901292	nucleoside phosphate catabolic process	biological process	0.04944957929	

GO	name	GO type	p-value FDR *D. melanogaster*	p-value FDR *D. virilis*
GO:0042302	structural constituent of cuticle	molecular function	1.05E-06	1.05E-04
GO:0042335	cuticle development	biological process	3.10E-09	
GO:0031012	extracellular matrix	cellular component	2.15E-08	
GO:0040003	chitin-based cuticle development	biological process	6.58E-08	
GO:0062129	chitin-based extracellular matrix	cellular component	6.84E-07	
GO:0005214	structural constituent of chitin-based cuticle	molecular function	1.05E-06	
GO:1901071	glucosamine-containing compound metabolic process	biological process	3.61E-04	
GO:0006040	amino sugar metabolic process	biological process	5.01E-04	
GO:0008010	structural constituent of chitin-based larval cuticle	molecular function	1.23E-03	
GO:0004099	chitin deacetylase activity	molecular function	1.39E-03	
GO:0048856	anatomical structure development	biological process	7.39E-03	
GO:0005198	structural molecule activity	molecular function	1.10E-02	
GO:0019213	deacetylase activity	molecular function	1.85E-02	
GO:0004252	serine-type endopeptidase activity	molecular function		2.53E-02
GO:0008236	serine-type peptidase activity	molecular function		3.58E-02
GO:0017171	serine hydrolase activity	molecular function		3.58E-02
GO:0005887	integral component of plasma membrane	cellular component	4.69E-02	

GO	name	GO type	p-value FDR *D. melanogaster*	p-value FDR *D. virilis*
GO:0005506	iron ion binding	molecular function		6.57E-06
GO:0016705	oxidoreductase activity, acting on paired donors, with incorporation or redu	molecular function		6.57E-06
GO:0020037	heme binding	molecular function		6.57E-06
GO:0046906	tetrapyrrole binding	molecular function		6.57E-06
GO:0016491	oxidoreductase activity	molecular function		6.73E-04
GO:0055114	oxidation-reduction process	biological process		1.04E-03
GO:0035080	heat shock-mediated polytene chromosome puffing	biological process	2.68E-02	
GO:0035079	polytene chromosome puffing	biological process	3.48E-02	

ST5. Late pupae-specific enriched GO terms

GO	name	GO type	p-value FDR *D. melanogaster*	p-value FDR *D. virilis*
GO:0042302	structural constituent of cuticle	molecular function		1.16E-09
GO:0031012	extracellular matrix	cellular component	4.36E-04	
GO:0005198	structural molecule activity	molecular function		3.07E-03
GO:0005887	integral component of plasma membrane	cellular component	1.19E-02	
GO:0008061	chitin binding	molecular function		1.48E-02
GO:0062129	chitin-based extracellular matrix	cellular component	2.11E-02	
GO:0031226	intrinsic component of plasma membrane	cellular component	2.55E-02	

GO	name	GO type	p-value FDR *D. melanogaster*	p-value FDR *D. virilis*
GO:0031966	mitochondrial membrane	cellular component	5.49E-03	6.19E-04
GO:0006090	pyruvate metabolic process	biological process	2.13E-02	7.94E-04
GO:0006165	nucleoside diphosphate phosphorylation	biological process	9.44E-03	2.28E-03
GO:0006096	glycolytic process	biological process	4.28E-03	6.58E-03
GO:0006757	ATP generation from ADP	biological process	4.28E-03	6.58E-03
GO:0046939	nucleotide phosphorylation	biological process	1.32E-02	2.28E-03
GO:0007601	visual perception	biological process	2.20E-02	2.28E-03
GO:0009135	purine nucleoside diphosphate metabolic process	biological process	9.79E-03	1.01E-02
GO:0009179	purine ribonucleoside diphosphate metabolic process	biological process	9.79E-03	1.01E-02
GO:0046031	ADP metabolic process	biological process	9.79E-03	1.01E-02
GO:0016052	carbohydrate catabolic process	biological process	1.28E-02	0.01013766833
GO:0009185	ribonucleoside diphosphate metabolic process	biological process	2.01E-02	1.01E-02
GO:0004743	pyruvate kinase activity	molecular function	3.00E-02	1.62E-02
GO:0030955	potassium ion binding	molecular function	3.00E-02	1.62E-02
GO:0031420	alkali metal ion binding	molecular function	3.00E-02	1.62E-02
GO:0032504	multicellular organism reproduction	biological process	2.95E-52	
GO:0000003	reproduction	biological process	3.23E-51	
GO:0005615	extracellular space	cellular component	1.09E-15	
GO:0003341	cilium movement	biological process	1.11E-09	
GO:0031514	motile cilium	cellular component	2.47E-09	
GO:0036126	sperm flagellum	cellular component	8.61E-08	
GO:0097729	9+2 motile cilium	cellular component	8.61E-08	
GO:0030286	dynein complex	cellular component	2.85E-07	
GO:0060294	cilium movement involved in cell motility	biological process	9.51E-07	
GO:0048029	monosaccharide binding	molecular function	5.32E-06	
GO:0019236	response to pheromone	biological process	6.05E-06	
GO:0005929	cilium	cellular component	6.22E-06	
GO:0005858	axonemal dynein complex	cellular component	6.72E-06	
GO:0001539	cilium or flagellum-dependent cell motility	biological process	7.93E-06	
GO:0060285	cilium-dependent cell motility	biological process	7.93E-06	
GO:0046008	regulation of female receptivity, post-mating	biological process	2.60E-05	
GO:0007602	phototransduction	biological process		3.15E-05

GO:0009581	detection of external stimulus	biological process		3.15E-05
GO:0009582	detection of abiotic stimulus	biological process		3.15E-05
GO:0009583	detection of light stimulus	biological process		3.15E-05
GO:0009605	response to external stimulus	biological process		3.15E-05
GO:0005975	carbohydrate metabolic process	biological process		3.33E-05
GO:0044703	multi-organism reproductive process	biological process	4.24E-05	
GO:0051704	multi-organism process	biological process	4.24E-05	
GO:0045434	negative regulation of female receptivity, post-mating	biological process	6.28E-05	
GO:0043695	detection of pheromone	biological process	7.61E-05	
GO:0044706	multi-multicellular organism process	biological process	7.86E-05	
GO:0046692	sperm competition	biological process	7.86E-05	
GO:0006099	tricarboxylic acid cycle	biological process	8.16E-05	
GO:0045924	regulation of female receptivity	biological process	8.16E-05	
GO:0030317	flagellated sperm motility	biological process	1.16E-04	
GO:0097722	sperm motility	biological process	1.16E-04	
GO:0007606	sensory perception of chemical stimulus	biological process	1.28E-04	
GO:0009628	response to abiotic stimulus	biological process		1.97E-04
GO:0009314	response to radiation	biological process		2.13E-04
GO:0009416	response to light stimulus	biological process		2.13E-04
GO:0016616	oxidoreductase activity, acting on the CH-OH group of donors, NAD or NA	molecular function		2.63E-04
GO:0015556	C4-dicarboxylate transmembrane transporter activity	molecular function	2.67E-04	
GO:0018095	protein polyglutamylation	biological process	3.11E-04	
GO:0016614	oxidoreductase activity, acting on CH-OH group of donors	molecular function		3.56E-04
GO:0007621	negative regulation of female receptivity	biological process	3.56E-04	
GO:0070286	axonemal dynein complex assembly	biological process	4.00E-04	
GO:0007600	sensory perception	biological process	4.99E-04	
GO:0070739	protein-glutamic acid ligase activity	molecular function	5.23E-04	
GO:0070740	tubulin-glutamic acid ligase activity	molecular function	5.23E-04	
GO:0055085	transmembrane transport	biological process	5.47E-04	
GO:0098656	anion transmembrane transport	biological process	5.72E-04	
GO:0018401	peptidyl-proline hydroxylation to 4-hydroxy-L-proline	biological process	6.93E-04	
GO:0004656	procollagen-proline 4-dioxygenase activity	molecular function	9.18E-04	
GO:0019798	procollagen-proline dioxygenase activity	molecular function	9.18E-04	

GO:0004867	serine-type endopeptidase inhibitor activity	molecular function	1.05E-03	
GO:0003998	acylphosphatase activity	molecular function		0.001074445715
GO:0016701	oxidoreductase activity, acting on single donors with incorporation of mole	molecular function	0.001138151711	
GO:0019511	peptidyl-proline hydroxylation	biological process	0.001174931459	
GO:0015141	succinate transmembrane transporter activity	molecular function	0.001540820312	
GO:0031545	peptidyl-proline 4-dioxygenase activity	molecular function	0.001540820312	
GO:0030246	carbohydrate binding	molecular function	0.001756309379	
GO:0050953	sensory perception of light stimulus	biological process		0.002277478376
GO:0060180	female mating behavior	biological process	0.002279700881	
GO:0005310	dicarboxylic acid transmembrane transporter activity	molecular function	0.002358207656	
GO:0031543	peptidyl-proline dioxygenase activity	molecular function	0.002552627972	
GO:0016702	oxidoreductase activity, acting on single donors with incorporation of mole	molecular function	0.002595857901	
GO:0045505	dynein intermediate chain binding	molecular function	0.002595857901	
GO:0006839	mitochondrial transport	biological process	0.002992801262	
GO:0015740	C4-dicarboxylate transport	biological process	0.003067219939	
GO:0051606	detection of stimulus	biological process		0.003197185802
GO:0015291	secondary active transmembrane transporter activity	molecular function	0.003376042937	
GO:0031418	L-ascorbic acid binding	molecular function	0.003519252682	
GO:0045504	dynein heavy chain binding	molecular function	0.004059195433	
GO:1990542	mitochondrial transmembrane transport	biological process	0.004315409788	
GO:0009132	nucleoside diphosphate metabolic process	biological process		0.005609414293
GO:0070735	protein-glycine ligase activity	molecular function	0.005832763966	
GO:0004457	lactate dehydrogenase activity	molecular function		0.005855681456
GO:0004459	L-lactate dehydrogenase activity	molecular function		0.005855681456
GO:0018126	protein hydroxylation	biological process	0.006173644484	
GO:1990204	oxidoreductase complex	cellular component		0.006260984624
GO:0015744	succinate transport	biological process	0.006548399453	
GO:0018094	protein polyglycylation	biological process	0.006548399453	
GO:0005342	organic acid transmembrane transporter activity	molecular function	0.007534349177	
GO:0046943	carboxylic acid transmembrane transporter activity	molecular function	0.007534349177	
GO:0015370	solute:sodium symporter activity	molecular function	0.008286116424	
GO:0005534	galactose binding	molecular function	0.008862267696	
GO:0070585	protein localization to mitochondrion	biological process	0.009127739366	

GO:0072655	establishment of protein localization to mitochondrion	biological process	0.009127739366	
GO:0015709	thiosulfate transport	biological process	0.009411630239	
GO:0015729	oxaloacetate transport	biological process	0.009411630239	
GO:0015743	malate transport	biological process	0.009411630239	
GO:0071422	succinate transmembrane transport	biological process	0.009411630239	
GO:0071423	malate transmembrane transport	biological process	0.009411630239	
GO:0030150	protein import into mitochondrial matrix	biological process	0.009438879178	
GO:0006835	dicarboxylic acid transport	biological process	0.01004178763	
GO:0005875	microtubule associated complex	cellular component	0.01081981585	
GO:0019200	carbohydrate kinase activity	molecular function	0.01092403046	
GO:0018208	peptidyl-proline modification	biological process	0.01137570845	
GO:1903825	organic acid transmembrane transport	biological process	0.01141927478	
GO:1905039	carboxylic acid transmembrane transport	biological process	0.01141927478	
GO:0005930	axoneme	cellular component	0.01159448831	
GO:0036157	outer dynein arm	cellular component	0.01163098088	
GO:0036158	outer dynein arm assembly	biological process	0.01262759041	
GO:0071482	cellular response to light stimulus	biological process	0.01359238018	
GO:0098800	inner mitochondrial membrane protein complex	cellular component	0.01367588853	
GO:0016491	oxidoreductase activity	molecular function		0.0162450171
GO:0098573	intrinsic component of mitochondrial membrane	cellular component	0.0168996024	
GO:0005343	organic acid:sodium symporter activity	molecular function	0.01708080832	
GO:0005506	iron ion binding	molecular function	0.01727608323	
GO:0015117	thiosulfate transmembrane transporter activity	molecular function	0.01842848004	
GO:0015131	oxaloacetate transmembrane transporter activity	molecular function	0.01842848004	
GO:0015140	malate transmembrane transporter activity	molecular function	0.01842848004	
GO:0036156	inner dynein arm	cellular component	0.01874540299	
GO:0015293	symporter activity	molecular function	0.01970413871	
GO:0005326	neurotransmitter transmembrane transporter activity	molecular function	0.01971606196	
GO:0008643	carbohydrate transport	biological process	0.02014684702	
GO:0045333	cellular respiration	biological process	0.02014684702	
GO:0005576	extracellular region	cellular component	0.02028527686	
GO:0007617	mating behavior	biological process	0.02109159141	
GO:0015849	organic acid transport	biological process	0.02175553457	

GO:0046942	carboxylic acid transport	biological process	0.02175553457	
GO:0009060	aerobic respiration	biological process	0.02261434586	
GO:0061135	endopeptidase regulator activity	molecular function	0.02311732195	
GO:0006817	phosphate ion transport	biological process	0.02349200894	
GO:0004396	hexokinase activity	molecular function	0.02418472997	
GO:0008865	fructokinase activity	molecular function	0.02418472997	
GO:0009055	electron transfer activity	molecular function	0.02418472997	
GO:0015175	neutral amino acid transmembrane transporter activity	molecular function	0.02418472997	
GO:0045503	dynein light chain binding	molecular function	0.02418472997	
GO:0007608	sensory perception of smell	biological process	0.02549526356	
GO:0015294	solute:cation symporter activity	molecular function	0.02616130439	
GO:0015144	carbohydrate transmembrane transporter activity	molecular function	0.02715304443	
GO:0022804	active transmembrane transporter activity	molecular function	0.02783061999	
GO:0004866	endopeptidase inhibitor activity	molecular function	0.02998259105	
GO:0019825	oxygen binding	molecular function	0.02998259105	
GO:0035082	axoneme assembly	biological process	0.03032641158	
GO:0061134	peptidase regulator activity	molecular function	0.03083962483	
GO:0035303	regulation of dephosphorylation	biological process		0.03088727878
GO:0030414	peptidase inhibitor activity	molecular function	0.03224527458	
GO:0005839	proteasome core complex	cellular component		0.03443734758
GO:0098803	respiratory chain complex	cellular component		0.03443734758
GO:0044743	protein transmembrane import into intracellular organelle	biological process	0.03478333172	
GO:0032592	integral component of mitochondrial membrane	cellular component	0.03622541158	
GO:0002027	regulation of heart rate	biological process	0.03654629174	
GO:0006123	mitochondrial electron transport, cytochrome c to oxygen	biological process	0.03654629174	
GO:0016059	deactivation of rhodopsin mediated signaling	biological process	0.03654629174	
GO:0019646	aerobic electron transport chain	biological process	0.03654629174	
GO:0035435	phosphate ion transmembrane transport	biological process	0.03654629174	
GO:0006102	isocitrate metabolic process	biological process	0.0406032645	
GO:0022857	transmembrane transporter activity	molecular function	0.04074344244	
GO:0005283	amino acid:sodium symporter activity	molecular function	0.04241459612	
GO:0008569	ATP-dependent microtubule motor activity, minus-end-directed	molecular function	0.04241459612	
GO:0004298	threonine-type endopeptidase activity	molecular function		0.04308418597

GO:0070003	threonine-type peptidase activity	molecular function		0.04308418597
GO:0030145	manganese ion binding	molecular function	0.04334717208	
GO:0044782	cilium organization	biological process	0.04391098746	
GO:0005744	TIM23 mitochondrial import inner membrane translocase complex	cellular component	0.04454098939	
GO:0051959	dynein light intermediate chain binding	molecular function	0.04488570713	
GO:0043648	dicarboxylic acid metabolic process	biological process	0.04613827247	
GO:0019098	reproductive behavior	biological process	0.04902730586	
